# Spliceosomal Prp8 intein at the crossroads of protein and RNA splicing

**DOI:** 10.1371/journal.pbio.3000104

**Published:** 2019-10-10

**Authors:** Cathleen M. Green, Zhong Li, Aaron D. Smith, Olga Novikova, Valjean R. Bacot-Davis, Fengshan Gao, Saiyang Hu, Nilesh K. Banavali, Dennis J. Thiele, Hongmin Li, Marlene Belfort

**Affiliations:** 1 Department of Biological Sciences and RNA Institute, University at Albany, Albany, New York, United States of America; 2 Division of Genetics, Wadsworth Center, New York State Department of Health, Albany, New York, United States of America; 3 Department of Pharmacology and Cancer Biology, Duke University School of Medicine, Durham, North Carolina, United States of America; 4 Division of Translational Medicine, Wadsworth Center, New York State Department of Health, Albany, New York, United States of America; 5 Department of Biomedical Sciences, School of Public Health, University at Albany, Albany, New York, United States of America; 6 Department of Molecular Genetics and Microbiology, Duke University School of Medicine, Durham, North Carolina, United States of America; 7 Department of Biochemistry, Duke University School of Medicine, Durham, North Carolina, United States of America; University of Melbourne, AUSTRALIA

## Abstract

The spliceosome is a large ribonucleoprotein complex that removes introns from pre-mRNAs. At its functional core lies the essential pre-mRNA processing factor 8 (Prp8) protein. Across diverse eukaryotes, this protein cofactor of RNA catalysis harbors a self-splicing element called an intein. Inteins in Prp8 are extremely pervasive and are found at 7 different sites in various species. Here, we focus on the Prp8 intein from *Cryptococcus neoformans* (*Cne*), a human fungal pathogen. We solved the crystal structure of this intein, revealing structural homology among protein splicing sequences in eukaryotes, including the Hedgehog C terminus. Working with the *Cne* Prp8 intein in a reporter assay, we find that the biologically relevant divalent metals copper and zinc inhibit intein splicing, albeit by 2 different mechanisms. Copper likely stimulates reversible modifications on a catalytically important cysteine, whereas zinc binds at the terminal asparagine and the same critical cysteine. Importantly, we also show that copper treatment inhibits Prp8 protein splicing in *Cne*. Lastly, an intein-containing Prp8 precursor model is presented, suggesting that metal-induced protein splicing inhibition would disturb function of both Prp8 and the spliceosome. These results indicate that Prp8 protein splicing can be modulated, with potential functional implications for the spliceosome.

## Abstract 2

A structural study shows that an intein interrupting the critical spliceosomal protein Prp8 in the fungal pathogen Cryptococcus neoformans is inhibited by biologically relevant metals, with implications for the regulation of RNA splicing.

## Introduction

The spliceosome is a massive ribonucleoprotein complex that performs intron splicing, an important process for maintaining genome diversity in eukaryotes. At the heart of the spliceosome is pre-mRNA processing factor 8 (Prp8), a large (approximately 270 kDa) and highly conserved protein [1]. Prp8 helps generate mature mRNA by coordinating critical rearrangements at the catalytic core of the spliceosome. This essential protein has been implicated in human disease [2,3], is evolutionarily linked to group II introns [4,5], and is structurally related to telomerase [6]. Recent advances in structural biology have shed new light onto both Prp8 and the spliceosomal machinery at atomic resolution, unveiling an unprecedented level of detail into the molecular steps of intron splicing [5,7–12].

A particular reason for our interest in Prp8 is that, across several organisms, this large protein contains a self-splicing intein at different positions, implying independent acquisition. Inteins are internal proteins that invade at the DNA level and undergo transcription and translation with the host gene [13–15]. The intein-containing precursor undergoes protein splicing, a process that excises the intein and ligates the flanking sequences, called exteins, to form the functional protein. Inteins are often bipartite, encoding a splicing domain for excision and ligation, and an endonuclease domain for homing [16,17]. Since some inteins are mobile, they are generally considered selfish genetic elements, but new research indicates that inteins can post-translationally regulate proteins [18–25].

Inteins are found in all 3 domains of life and are especially abundant in bacteria and archaea [26]. In eukaryotes, inteins are sparse but have been found in nuclear and chloroplast genomes with distinct patterns of insertion [27]. Nuclear inteins tend to be in proteins that are involved in energy metabolism and RNA processing, whereas chloroplast inteins are found in proteins that carry out transcription and replication. Out of all the intein-harboring proteins in eukaryotes, Prp8 is overwhelmingly favored. There are over 100 inteins identified across various sites of Prp8 in different species.

Pathogenic fungi seem to be enriched for inteins [27,28]. Several notable human pathogens contain Prp8 inteins, including *Aspergillus fumigatus*, *Histoplasma capsulatum*, and *Cryptococcus neoformans* (*Cne*). Intriguingly, many organisms with Prp8 inteins also tend to be intron-rich [29]. The presence of inteins in Prp8 and the correlation with intron density beg the question of an intein benefit to the host and especially to pathogens. To begin to answer this question, we focus on the Prp8 intein from *Cne*. This is a mini-intein, naturally lacking the homing endonuclease domain, at only 171 amino acid residues. The intein is also found at a highly conserved site at the center of Prp8 and thus is at the core of the spliceosome [1,5,30].

Studying the Prp8 intein present in *Cne* addresses questions of conditional protein splicing in an important human pathogen in an entirely new domain of life. Solving the Prp8 intein structure set the stage for beginning such studies and provided evolutionary context by revealing similarities to the metazoan Hedgehog protein. Biochemical experiments then showed that the *Cne* Prp8 intein is differentially responsive in vitro to copper and zinc, metals encountered by pathogens in immune cells during infection. Importantly, copper also showed protein splicing inhibition in vivo in *Cne*, the native host. Further, creation of a Prp8 precursor model illustrates how intein presence relates to the native protein and hints at how the intein could influence both Prp8 function and spliceosome assembly.

## Results

### Prp8 is an intein hot spot with diverse insertion sites

**Fig 1:**
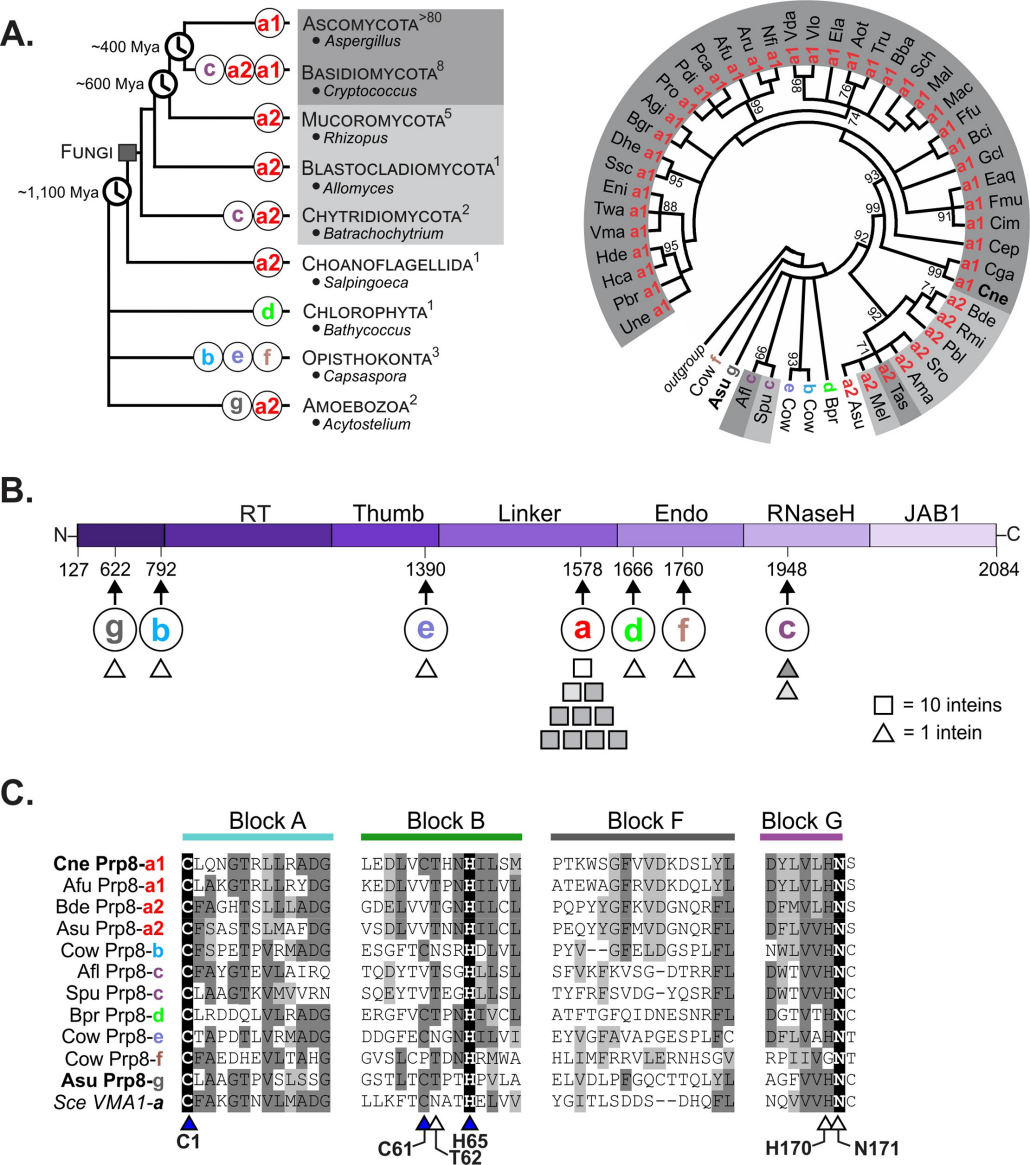
Prp8 is an intein hot spot with multiple, independent insertion sites. (A) Modified phylogenetic tree of eukaryotes (left) shows the phyla that contain Prp8 inteins, with representative genera listed. Evolutionary divergence times are denoted in mya. The number of Prp8 inteins in each phylum is shown in superscript, and insertion sites are shown on the branches (colored a–g). A phylogenetic intein tree (right) was reconstructed based on the amino acid sequences of intein splicing blocks for a subset of 50 Prp8 inteins. The radial tree shows numerous clusters, which correspond to grouping by insertion site. Abbreviated species names are shown (full names in S1 Fig). Shading (dark gray, light gray, or white) indicates phylogenetic distribution. The divergence of the inteins despite extein conservation (S4 Fig) suggests independent invasions. (B) A line diagram of the exteins (amino acid residues 127 to 2084) shows the domains of the Prp8 protein. The arrows below indicate the site of intein insertion (a–g) with the corresponding residue number based on *Saccharomyces cerevisiae* Prp8 (PDB 5GMK, chain A). Shapes represent how many inteins are found at each site (square = 10 inteins, triangle = 1 intein) and are shaded to denote phylogenetic origin as in Fig 1A. Prp8-a is the most common insertion site with approximately 100 inteins. (C) Multiple sequence alignment of the splicing blocks of Prp8 inteins from each insertion site. Comparative analysis of residues found in Blocks A, B, F, and G reveals that Prp8 inteins occupying other insertion sites are substantially different from one another, indicating independent acquisition. Identical residues are critical to self-splicing. Triangles indicate residues of general interest and those shaded blue are of specific interest. Numbers correspond to the *Cne* Prp8 intein. Shading is as follows: black, identical amino acid; dark gray, conserved amino acid; light gray, similar amino acid substitution. *Cne*, *Cryptococcus neoformans*; mya, millions of years ago; PDB, Protein Data Bank; Prp8, pre-mRNA processing factor 8.

Recent data mining revealed over 100 inteins in the Prp8 protein present across assorted eukaryotic groups, some of which emerged as far back as approximately 1,100 million years ago (mya; Fig 1A, left) [27,31–33]. The vast majority of Prp8 inteins are found across different fungal species, particularly in Ascomycota, and the rest are dispersed in other eukaryotic phyla (Fig 1A, left). To characterize these Prp8 inteins, we performed comparative and phylogenetic analyses on a representative subset based on the splicing motifs (Fig 1; S1–S4 Figs) [15,34]. In total, there are 7 distinct intein insertion points, denoted Prp8-a through Prp8-g (Fig 1; S1–S4 Figs) [33,35,36]. With only a few exceptions, fungal Prp8 inteins occupy the same insertion site, Prp8-a (Fig 1A and 1B) [27,31,33]. The Prp8-g insertion is reported here for the first time (Fig 1B) and was found at the N-terminal end of Prp8 in the social amoeba *Acytostelium subglobosum* [37] (S3 Fig).

The reconstructed phylogenetic tree reveals that Prp8 inteins group by insertion site (Fig 1A, right; S1 Fig). Although insertion sites b through g have limited representation, the observed clustering, as well as the level of sequence divergence between inteins from different insertion sites, suggest multiple independent intein invasion events throughout evolutionary history. Importantly, bifurcation of Prp8-a inteins into 2 well-supported clusters (a1 and a2, interior-branch test value of 92%) indicates recurrent invasion of inteins into site a across diverse fungi (S1A Fig). Furthermore, Prp8 extein phylogenetic analyses show clustering by host organism, adding support to independent intein acquisitions (S4 Fig). All 7 insertion sites were mapped to a simplified line diagram of Prp8 exteins and are peppered across the various domains (Fig 1B).

A multiple sequence alignment of the intein splicing motifs, referred to as Blocks A, B, F, and G, demonstrates the sequence divergence among Prp8 inteins (Fig 1C; S2 Fig). Other than identical residues located in Blocks A, B, and G (Fig 1C, black shading; S2 Fig), Prp8 inteins from different insertion sites share limited sequence homology. Block A contains the first residue of the intein, a highly conserved cysteine called C1, which performs the first nucleophilic attack of the protein splicing pathway. This amino acid is identical across the disparate Prp8 inteins (Fig 1C, Block A; S2 Fig). Block B usually carries a highly conserved motif known as TxxH [38]. Across the Prp8 inteins, Block B histidine of TxxH is present in all analyzed inteins, whereas the threonine is not as conserved (Fig 1C, Block B; S2 Fig). Also alike across all Prp8 inteins is a terminal asparagine at the C terminus of the intein in Block G, which also directly participates in splicing (Fig 1C, Block G; S2 Fig). The first amino acid of the C extein, known as the +1 residue, is usually a cysteine, serine, or threonine, and all Prp8 inteins use one of these as the +1 nucleophile. Block F shows little conservation across Prp8 inteins. The *Saccharomyces cerevisiae* (*Sce*) Vma1 intein, in the vacuolar ATPase, is as similar to Prp8 inteins as other Prp8 inteins are to each other (Fig 1C), indicating a close ancestral relationship. The poor sequence alignment among Prp8 inteins reinforces that distinct inteins recurrently invaded Prp8.

### *Cne* Prp8 intein structure shows similarity to eukaryotic protein splicing elements

**Fig 2:**
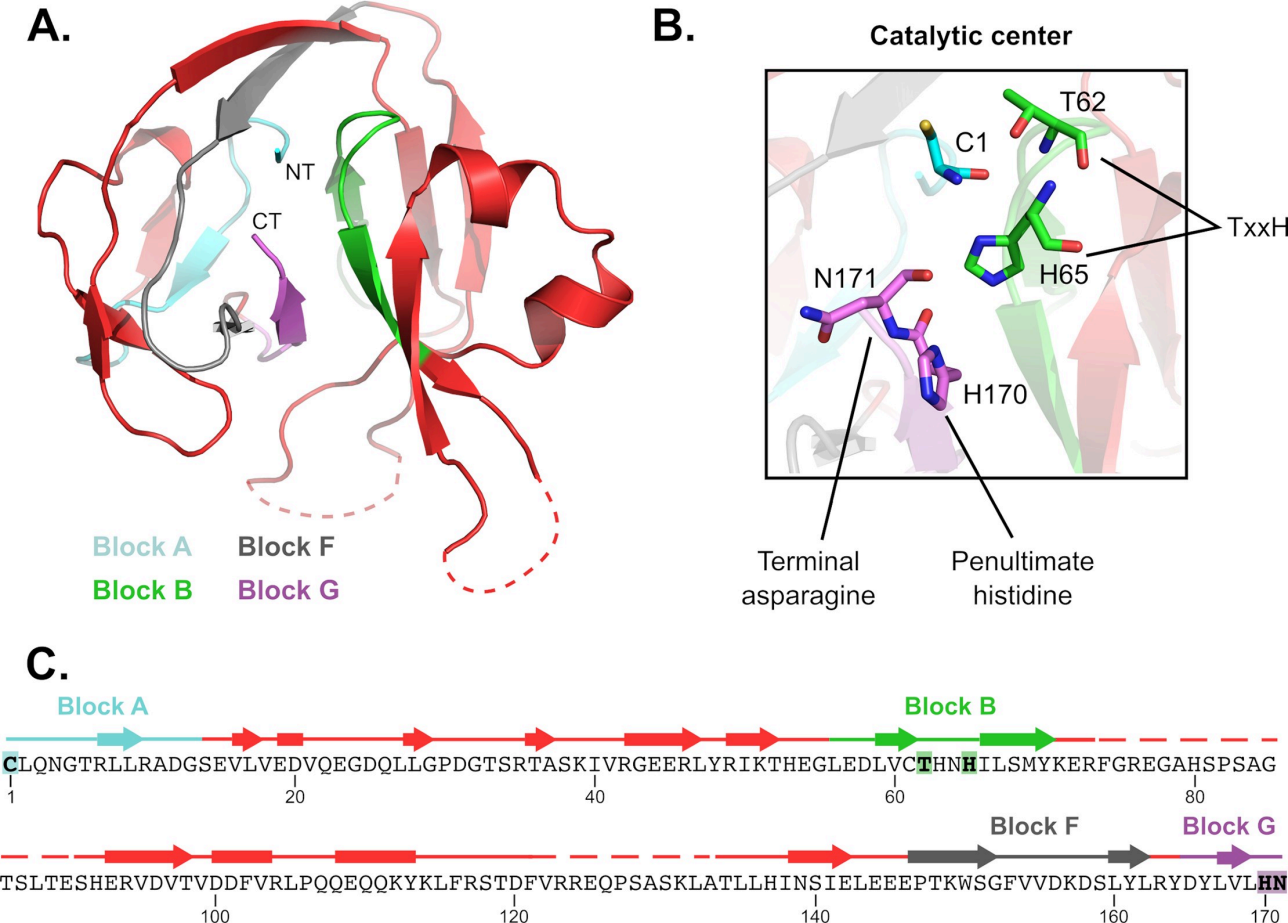
Structure and features of the *Cne* Prp8 intein. (A) A crystal structure of the *Cne* Prp8 intein from site a was solved to 1.75 Å resolution. This structure has the canonical horseshoe shape and resolution of the 4 splicing blocks, indicated in cyan (Block A), green (Block B), gray (Block F), and purple (Block G; Fig 1C). The NT and CT are annotated. Some regions of the structure are unresolved (dashed lines) and likely represent remnants of the original endonuclease domain or linker sequences. (B) Features of the active site. The catalytic center is shown, highlighting the first residue of the intein (C1), the penultimate histidine (H170), and the terminal asparagine (N171). The Block B TxxH motif is shown in green with T62 and H65 represented as sticks. These residues are critical to carrying out autocatalytic protein splicing. (C) Sequence of the *Cne* Prp8 intein (residues 1–171) overlaid with secondary structure features. Blocks distant in sequence fold close in 3D space to allow protein splicing to occur. Unresolved regions (dashed lines) are between Blocks B and F. Arrows represent β-strands, rectangles are α-helices. Residues noted in Fig 2B are highlighted. *Cne*, *C*. *neoformans*; CT, carboxy terminus; NT, amino terminus; Prp8, pre-mRNA processing factor 8.

We next solved the crystal structure of the *Cne* Prp8 intein found at site a (Fig 2A; S4A Table). This intein was chosen because of its small size and because it is found in a significant human pathogen. The *Cne* Prp8 intein was solved to 1.75 Å resolution (Fig 2A). This novel structure helped us develop a sense of structural relatedness of the *Cne* Prp8 intein to other inteins and intein-like elements and to later model the intein into both the Prp8 protein and the spliceosome.

The *Cne* Prp8 intein structure represents only the second known fungal nuclear intein structure. The first was of the *Sce* Vma1 intein, which was solved with its linker domain, a connector between the splicing blocks and the internal endonuclease domain [39,40]. As with all solved intein structures so far, the *Cne* Prp8 intein has the canonical horseshoe shape, created by pseudo–2-fold symmetry that positions the catalytic N- and C termini in close proximity (Fig 2A). Highlighting the splicing blocks (A, B, F, and G), we see the active core that carries out autocatalytic protein splicing (Fig 2A and 2B) [15,34]. This catalytic center contains the residues essential for splicing: the nucleophilic cysteine (C1) in Block A, and the terminal asparagine (N171) in Block G (Fig 2B and 2C; S5 Fig). The C1 and N171 are also positioned in the vicinity of the conserved Block B TxxH residues (T62 and H65), which are important for priming the intein for self-excision at its amino terminus (Fig 2B) [41]. All of these residues contribute critically to the protein splicing pathway, which involves a series of nucleophilic attacks, cyclization of the terminal asparagine, and reformation of a peptide bond between the exteins to form the functional protein [42]. Overlaying the *Cne* Prp8 intein primary sequence with its secondary structure shows the position of residues from each block within the context of the 3D architecture (Fig 2C). For example, Blocks A and B are far apart in sequence but fold proximally in 3D space to execute protein splicing (Fig 2). This representation also illustrates that the unresolved regions of the intein are between Block B and Block F and likely represent flexible linker sequences of a former endonuclease domain (Fig 2A and 2C).

**Fig 3:**
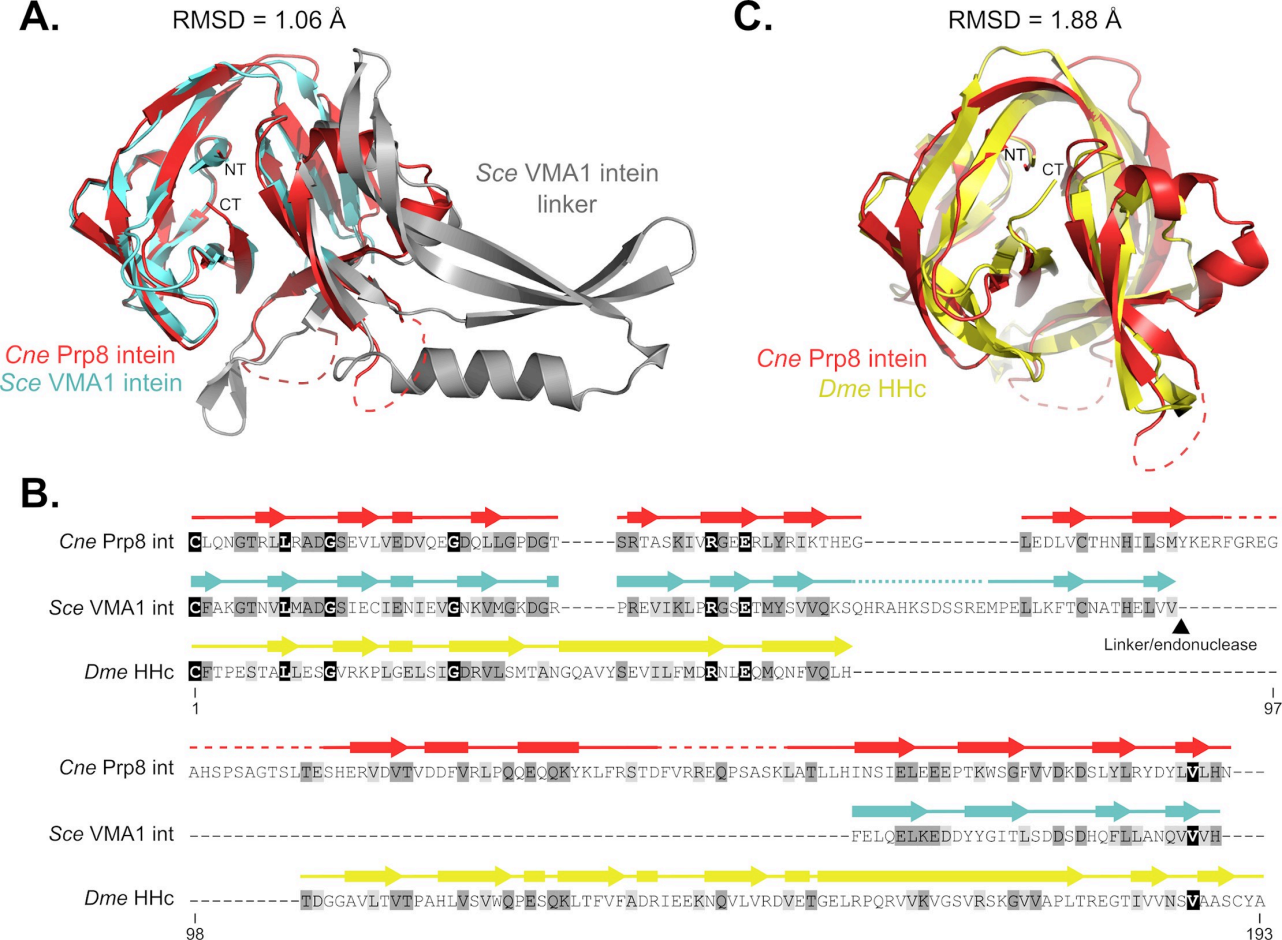
Similarity of the *Cne* Prp8 intein to eukaryotic protein splicing elements. (A) Overlay of the *Cne* Prp8 intein (red) with the *Sce* Vma1 intein (cyan and gray, PDB 1GPP) with an RMSD of 1.06 Å. Structural similarities are most pronounced in the splicing domain (Blocks A, B, F, and G, cyan) of the *Sce* Vma1 intein, whereas the linker/endonuclease domain (gray) is where the *Cne* Prp8 intein did not resolve (dashed lines). The NT and CT are annotated. (B) Multiple sequence alignment of *Cne* Prp8 intein, *Sce* Vma1 intein, and *Dme* HHc. Sequence comparison (residues 1–193) reveals significant differences across the 3 proteins, which are only 22.6% identical. Overlaying secondary structure shows that, despite sequence divergence, the proteins have high structural similarity. Shading is as follows: black, identical amino acid; dark gray, conserved amino acid; light gray, similar amino acid substitution. Arrows represent β-strands, rectangles are α-helices. Unresolved regions shown as dashed lines. (C) Overlay of the *Cne* Prp8 intein (red) with *Dme* HHc (yellow, PDB 1AT0). Structural 3D BLAST shows parallels between the eukaryotic intein and the eukaryotic protein splicing Hedgehog domain, with an RMSD of 1.88 Å. The NT and CT are annotated. *Cne*, *C*. *neoformans*; CT, carboxy terminus; *Dme*, *Drosophila melanogaster*; HHc, Hedgehog C-terminal domain; NT, amino terminus; PDB, Protein Data Bank; Prp8, pre-mRNA processing factor 8; RMSD, Root-mean-square deviation; *Sce*, *Saccharomyces cerevisiae*.

We next performed a 3D BLAST to compare the *Cne* Prp8 intein structure to other solved structures (Fig 3). Unsurprisingly, the *Cne* Prp8 intein structure as the query returns the *Sce* Vma1 intein as the top hit (Fig 3A, PDB 1GPP) [39]. These are both fungal inteins encoded in nuclear genomes. An overlay of the *Cne* Prp8 intein (red) and the *Sce* Vma1 intein (splicing domain in cyan, linker/endonuclease domain in gray) displays high structural similarity in the splicing modules (Fig 3A, Root-mean-square deviation [RMSD] of 1.06 Å). The unstructured regions in the *Cne* Prp8 intein structure are where the *Sce* Vma1 intein encodes a linker domain (Fig 3, dashed red lines). A closer look at the active centers of the *Cne* Prp8 intein and the *Sce* Vma1 intein demonstrates unmistakable overlap of the catalytic residues (S5A Fig), further confirming the similarities.

Another top hit from the 3D BLAST is the 17 kDa fragment of the *Drosophila melanogaster* (*Dme*) Hedgehog C-terminal domain (HHc; Fig 3B and 3C) [43]. Hedgehog is an essential signaling molecule in higher eukaryotes with an analogous cleavage reaction performed by a highly conserved cysteine [44]. This allows the N-terminal domain of Hedgehog to ligate to a cholesterol molecule, which plays a critical role in metazoan development. There has been considerable speculation about the relatedness of Hedgehog and inteins [43,45]. It was recently proposed, based on sequence similarity, that the N-terminal portion of Hedgehog was acquired through horizontal gene transfer from a prokaryote [46]. However, a sequence alignment between the *Cne* Prp8 intein, the *Sce* Vma1 intein, and *Dme* HHc shows only an average of 22.6% sequence identity (Fig 3B, 26.4% identity Vma1 to Prp8, 19.2% HHc to Prp8, 22.2% Vma1 to HHc). One highly conserved residue across the 3 proteins is the initiating cysteine, shared by all the sequences, as well as a C-terminal valine (Fig 3B, black shading). Despite the sequence divergence, a secondary structure overlay demonstrates that these sequences all code for the same structural elements (Fig 3B). The *Cne* Prp8 intein and *Dme* HHc have an RMSD of 1.88 Å (Fig 3C, PDB 1AT0), sharing a similar degree of structural relatedness as a bacterial and a fungal intein (S5B Fig, RMSD 2.22 Å, PDB 2IMZ) [47]. These results reinforce the evolutionary connection between inteins and Hedgehog proteins.

### *Cne* Prp8 intein is responsive to stress

**Fig 4:**
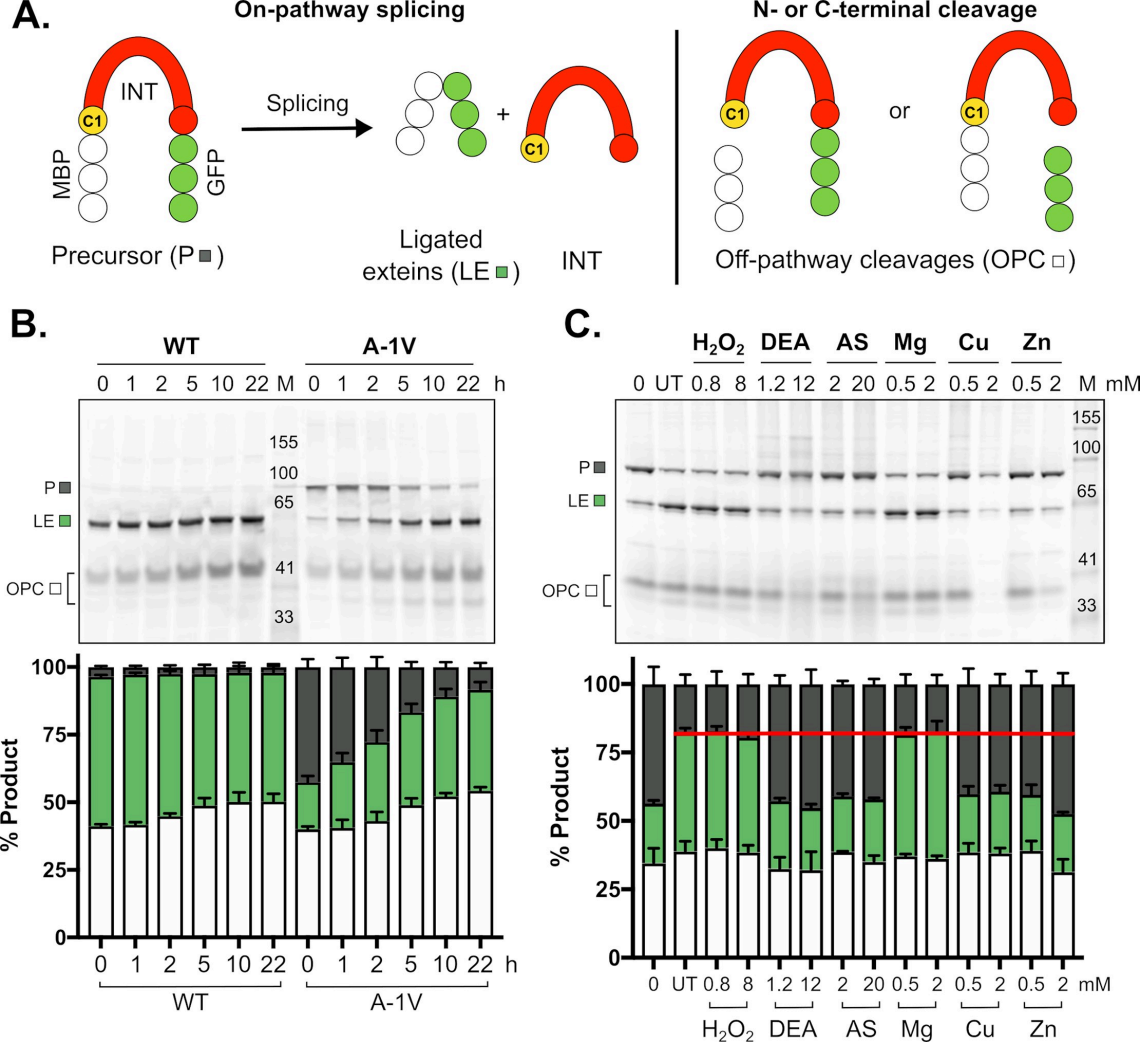
MIG Prp8 A-1V is responsive to metal and RNS treatment. (A) Schematic of the MIG reporter. The construct contains MIG and is expressed in *E*. *coli*. GFP allows monitoring of splicing using in-gel fluorescence. The P can undergo protein splicing (left), yielding LE and free INT (not seen on gels). OPC reactions (right), such as N- and C-terminal cleavage, can also occur. The catalytic cysteine, C1, is labeled. (B) MIG Prp8 WT splices rapidly. A fluorescent gel of a splicing time course shows that the WT *Cne* Prp8 intein in MIG is entirely spliced by the start of the assay (left, WT, 0 h). The A-1V mutant had precursor at the assay start and spliced over time (right, A-1V, 0 h). Quantitation is shown below in stacked plots. Data are representative of 3 biological replicates and mean standard deviations are shown. Data available in S1 Data. (C) MIG Prp8 A-1V accumulates precursor under RNS and metal treatment. After 5 h in vitro treatment with a panel of environmental stressors, there was an increase in P compared to the UT (red line) with the RNS compounds DEA at 1.2 mM and 12 mM and AS at 2 mM and 20 mM, and the metals Cu and Zn at 0.5 mM and 2 mM. H_2_O_2_ and Mg showed no effect at either concentration. Quantitation is shown below in a stacked plot. Data are representative of 3 biological replicates and mean standard deviations are shown. Data available in S1 Data. AS, Angeli’s salt; *Cne*, *C*. *neoformans*; DEA, DEA NONOate; GFP, green fluorescent protein; INT, intein; LE, ligated exteins; MBP, maltose-binding protein; MIG, MBP-Intein-GFP; OPC, off-pathway cleavage; P, precursor; Prp8, pre-mRNA processing factor 8; RNS, reactive nitrogen species; UT, untreated; WT, wild type.

With the structure solved, we next sought to investigate Prp8 intein splicing and whether it is regulated in any way. For simplicity, the *Cne* Prp8 intein was studied in *Escherichia coli*. Given that full-length Prp8 contains approximately 2,500 amino acids, we cloned the *Cne* Prp8 intein into a reporter construct that uses maltose binding protein (MBP) and green fluorescent protein (GFP) as foreign N and C exteins, respectively [21,24] (Fig 4A). From this construct, termed MBP-Intein-GFP (MIG), which contains 5 native N- and C-extein residues, expression is induced and splicing products, such as ligated exteins (Fig 4A, LE), are visualized using nonreducing SDS-PAGE and scanning for GFP fluorescence (Fig 4A, left). Off-pathway cleavage (OPC) products, the result of either N-terminal or C-terminal cleavage, are also detectable in the gels. N-terminal cleavage occurs when the thioester bond generated by the first step of protein splicing is cleaved by an external nucleophile. C-terminal cleavage is caused when the terminal asparagine (N171) cyclizes prior to the first step of protein splicing (Fig 4A, right).

First, we observed that the *Cne* Prp8 intein splices well in the foreign context to yield ligated exteins, as has been published previously by Pearl and colleagues [48]. However, splicing was so rapid that the amount of precursor remaining after induction (0 h) did not provide a suitable dynamic range for performing splicing assays (Fig 4B, WT). To slow down splicing and accumulate precursor, a mutation was made to the last residue of the N extein (referred to as -1), a site previously shown to affect splicing rates [49]. After random mutagenesis, a slower splicing mutant was isolated (Fig 4B, A-1V). The MIG Prp8 A-1V mutant has 40% precursor at 0 h and is splicing active over time (Fig 4B, A-1V). Interestingly, an A-1V mutant of the *Cne* Prp8 intein was previously shown to have attenuated splicing in other extein contexts [48], suggesting that splicing is somewhat dependent on both local flanking residues and distant extein context. It is worth noting that splicing rates are also intein-dependent, given that other Prp8-a inteins from fungal pathogens exhibit diverse splicing phenotypes when cloned into MIG (S6 Fig).

Next, using MIG Prp8 A-1V, we asked whether a condition exists in which intein splicing might be regulated. Treatments chosen were to mimic environmental stress that *Cne* experiences during infection, such as reactive oxygen species (ROS), reactive nitrogen species (RNS), and metals, all of which prevail during the intracellular respiratory burst (Fig 4C) [50,51]. From this initial panel, the RNS compounds DEA NONOate and Angeli’s salt showed significant precursor accumulation (Fig 4C, DEA and AS). It also appears that copper and zinc can cause splicing inhibition of MIG Prp8 A-1V (Fig 4C, Cu and Zn). Under these conditions, splicing was inhibited by approximately 50% (Fig 4C). This preliminary compound screen indicates that the *Cne* Prp8 intein may be subject to inhibition by specific conditions that occur during infection.

### Splicing inhibition is mechanistically distinct under copper and zinc treatment

**Fig 5:**
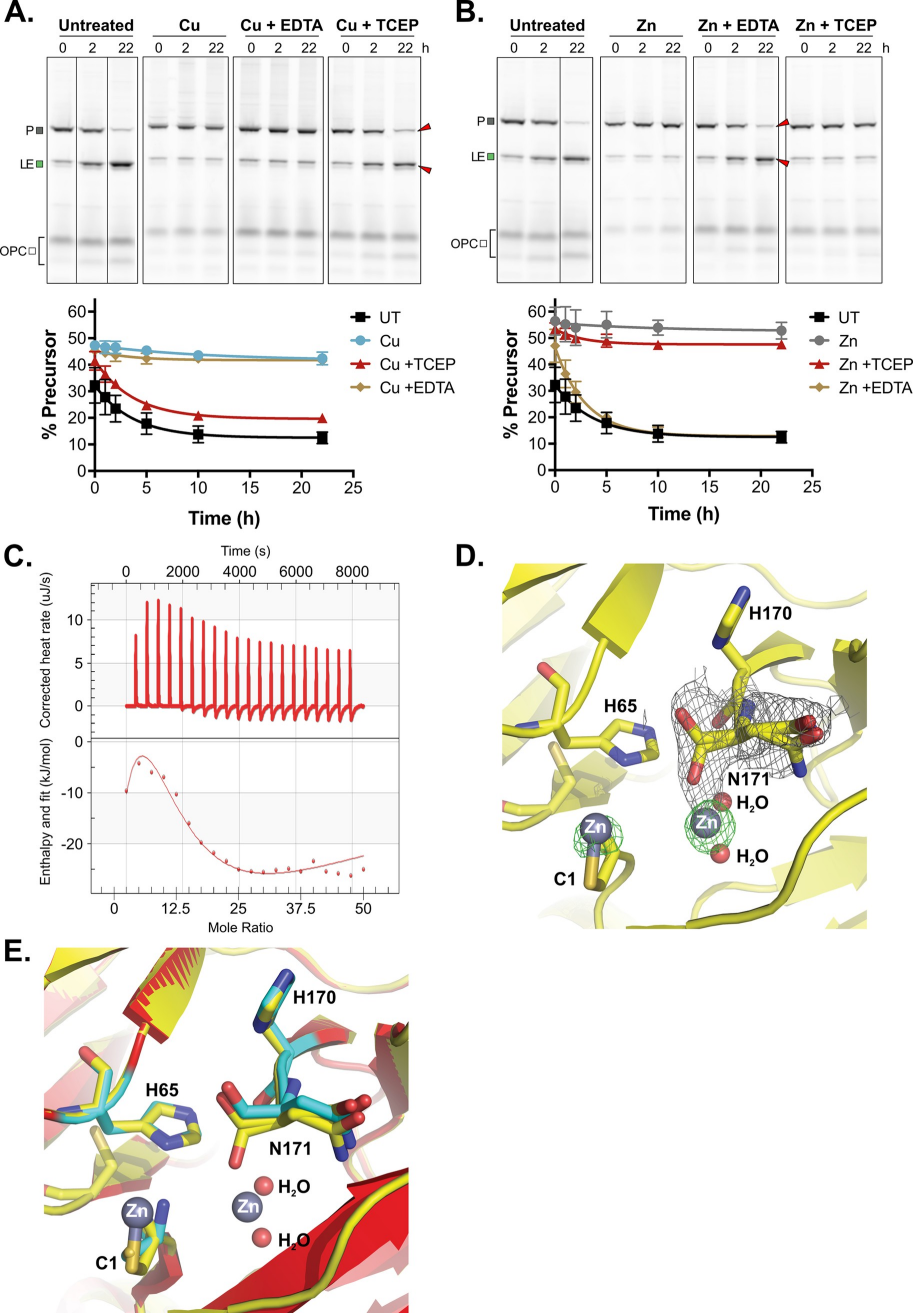
MIG Prp8 A-1V is differentially inhibited by copper and zinc. (A) Copper inhibition is alleviated by reducing agent only. MIG Prp8 A-1V splicing was completely inhibited by 1 mM copper treatment (Cu) over 22 h, given minimal loss in P or increase in LE occurred compared with the UT control. The inhibition was unaffected by treatment with metal chelator EDTA at 10 mM (Cu + EDTA). Upon adding copper for 2 h and then reducing agent TCEP at 40 mM (Cu + TCEP), splicing was restored and P converted into LE over time. Red arrows indicate splicing rescue. The splice products were quantitated, and the percent precursor is plotted as a proxy for splicing inhibition. Representative gels are shown. Data are representative of 3 biological replicates and mean standard deviations are shown. Data available in S1 Data. Lines through gels indicate where intervening lanes were cropped out of the image. (B) Zinc treatment is relieved by EDTA only. MIG Prp8 A-1V splicing was strongly inhibited by 1 mM zinc treatment (Zn) over 22 h compared with UT lysates. The zinc-based inhibition was relieved when treated with 10 mM EDTA (Zn + EDTA) after 2 h of zinc treatment, and splicing was observed at a rate comparable with the untreated samples. Red arrows indicate splicing rescue. When adding zinc for 2 h and then reducing agent TCEP at 40 mM (Zn + TCEP), splicing was unaffected. Representative gels are shown. Data are representative of 3 biological replicates and mean standard deviations are shown. Data available in S1 Data. Lines through gels indicate where intervening lanes were cropped out of the image. (C) Zinc binds to the *Cne* Prp8 intein tightly. Using ITC, 16 μM purified *Cne* Prp8 intein was titrated with 0.05 mM ZnSO_4_ over 20 injections at 37°C and pH 7.0 on a Nano ITC. The binding isotherm (bottom) shows integrated heat per mole of ZnSO_4_ as a function of the molar ratio of ZnSO_4_ to the *Cne* Prp8 intein and a K_d_ of 1 ± 0.82 nM was calculated. The NanoAnalyze ITC software automatically discarded outlier data points. Experiment was performed in triplicate. (D) Two binding sites in the *Cne* Prp8 intein-Zn^2+^ crystal structure. A close-up view of the crystal structure of the *Cne* Prp8 intein soaked with zinc shows 2 densities, one surrounding the terminal asparagine (N171) at the C terminus and one around the catalytic cysteine (C1), at the N terminus. Electron density maps are shown for the bound Zn^2+^ with an omit Fo-Fc difference map (green mesh) contoured at 5δ level and for the alternative conformation of N171 with a 2Fo-Fc map (gray mesh) contoured at 1δ level. Atomic colors are as follows: oxygen, red; carbon, yellow; nitrogen, blue; Zn^2+^, gray. Zn^2+^ and water molecules are shown as spheres, and the *Cne* Prp8 intein residues at the binding site are represented as sticks. (E) Minor conformational changes in the zinc-bound *Cne* Prp8 intein. A structural superimposition is shown of the native Prp8 intein (red) and the Prp8-Zn^2+^ complex (yellow) at the Zn^2+^ binding sites. Atomic colors are as in panel D, except that the carbon atoms for residues C1, H65, H170, and N171 of the native structure are in cyan. *Cne*, *C*. *neoformans*; EDTA, ethylenediaminetetraacetic acid; ITC, isothermal titration calorimetry; LE, ligated exteins; MIG, MBP-Intein-GFP; P, precursor; Prp8, pre-mRNA processing factor 8; TCEP, tris-(2-carboxyethyl)phosphine; UT, untreated.

Metal binding has been reported for other inteins and often engages catalytic residues, which would stall protein splicing [47,52–57]. Therefore, we chose to follow up on the observed copper and zinc inhibition by running in vitro MIG Prp8 A-1V assays to further assess effects on protein splicing over time (Fig 5; S7 and S8 Figs). Because higher concentrations of copper can cause protein precipitation (Fig 4C), we decreased each metal to 1 mM.

We found that 1 mM CuSO_4_ caused strong splicing inhibition for up to 22 h compared with untreated controls (Fig 5A, Untreated and Cu; S7 Fig). This inhibition persisted up to 30 h (S7A Fig). To test a copper-binding hypothesis, the same assay was carried out, but after 2 h of incubation with copper, ethylenediaminetetraacetic acid (EDTA) was added in excess to the remaining copper-treated lysate. EDTA chelates copper and should strip bound copper from the *Cne* Prp8 intein so that splicing can occur. However, addition of EDTA did not rescue splicing, ruling out the possibility of inhibition purely by copper binding (Fig 5A, Cu + EDTA).

Copper is a redox active metal that can cause cysteine oxidation, either by promoting disulfide bond formation or by catalyzing reversible or irreversible oxidative modifications [58]. We next tested whether the *Cne* Prp8 intein cysteines are being reversibly modified by copper, which would prevent the C1 from performing the first nucleophilic attack, and has precedent in intein biology [21,25]. We added the reducing agent tris-(2-carboxyethyl)phosphine (TCEP) to copper-treated lysate after collecting a sample after 2 h incubation. Strikingly, TCEP completely relieved the splicing inhibition (Fig 5A, Cu + TCEP). After reduction, MIG Prp8 A-1V precursor conversion into ligated exteins occurred at a rate similar to that of no copper treatment (Fig 5A, bottom), indicating reversible cysteine oxidation. TCEP can reduce copper, which could also lead to the loss of inhibition.

The *Cne* Prp8 intein only has 2 cysteines: C1 in Block A, and C61 in Block B, immediately preceding the TxxH motif (see Fig 1C, blue arrowheads). The C1 to C61 distance is 8.9 Å (S7B Fig), generally too far to form a disulfide bond [21,59,60]. We also found that C61 is not conserved among known Prp8 inteins (Fig 1C; S2 and S7C Figs), and the most commonly used residue at this site is valine (S2 Fig). Therefore, to investigate if C1 modifications are sufficient to inhibit protein splicing, several mutants of C61 in MIG Prp8 A-1V were tested for splicing activity (S8A Fig) and treated with copper (S8B Fig). The C61 mutants also showed precursor accumulation with copper treatment (S8B Fig), suggesting that C1-C61 disulfide bonding is not the underlying inhibitory mechanism, and that copper induces at least C1 oxidation, which is enough to cause the nonsplicing phenotype.

We further confirmed cysteine modification by performing mass spectrometry on purified *Cne* Prp8 intein. This showed a peak shifted by 32 Da, consistent with an addition of 2 oxygen atoms (S9A Fig). Additional validation pinpointed reversible sulfenic acid modifications (-SOH) to C1 and C61 with copper treatment (S9B Fig), but these were also present in the untreated *Cne* Prp8 intein (S9A Fig). This indicates that the *Cne* Prp8 intein has highly reactive cysteines that can be modified by atmospheric oxygen alone. Such extreme sensitivity has been observed for other inteins that are regulated by cysteine modification [21]. At this time, it is unclear whether the modifications in this assay are the result of copper, oxygen in air, or both. Based on our MIG data, reversible, copper-induced cysteine modifications are the most likely explanation for the inhibition we observe (Fig 5A) and are mediated mainly through C1 (S8B Fig).

Next, zinc, a metal without redox activity, was added to MIG Prp8 A-1V lysates given that it too was inhibitory in preliminary treatments (Fig 4C). The addition of 1 mM ZnSO_4_ also caused protein splicing inhibition and for similar time periods (Fig 5B, Untreated and Zn). To probe the mechanism of zinc inhibition, we followed up with the same EDTA chelation and TCEP reduction after taking samples treated with zinc for 2 h. In contrast to copper, EDTA alleviated protein splicing inhibition with zinc (Fig 5B, Zn + EDTA), but TCEP reduction did not (Fig 5B, Zn + TCEP). Thus, zinc likely causes inhibition by binding to the *Cne* Prp8 intein, because it is redox inactive and TCEP treatment yielded no change.

To corroborate zinc binding, purified *Cne* Prp8 intein was titrated with zinc in an isothermal titration calorimetry (ITC) experiment. This revealed tight binding of zinc to the intein, with a K_d_ in the 1 nM range (Fig 5C). To further understand the mode of zinc binding, we turned to crystal soaking in zinc acetate (S4B Table). We determined the crystal structure of the *Cne* Prp8 intein in complex with Zn^2+^ at 1.85 Å resolution (Fig 5D and 5E). Compared with the native form, the complex does not show significant conformational changes, as is reflected by a small overall RMSD (0.17 Å) between the 2 structures (Fig 5E). There are 6 molecules per asymmetric unit cell. All 6 molecules bind 1 Zn^2+^ at their C terminus with the terminal asparagine (N171), and 3 molecules bind an additional Zn^2+^ at their N terminal C1 (Fig 5D). It is currently unclear why all 6 molecules do not bind a second Zn^2+^ at C1. It is possible that the second zinc site, coordinated by the C1 side chain and main chain amide, has a lower affinity, and the high concentration of Zn^2+^ (4 mM) used for soaking allowed partial binding. Interestingly, a dissociated platinum atom binds to the C1 in a similar fashion in the mycobacterial RecA intein [52]. The Zn^2+^ at the C terminus is coordinated by the main chain carboxyl oxygen of the N171 and 2 water molecules. Binding at this site induces an alternative conformation for the main chain peptide carboxyl group to provide a ligand for Zn^2+^. This alternative conformation is not seen in the native structure (Fig 5D and 5E). Zn^2+^ binding to either of these 2 catalytic residues prevents Prp8 protein splicing.

### Protein splicing in *Cne* is inhibited by copper

**Fig 6:**
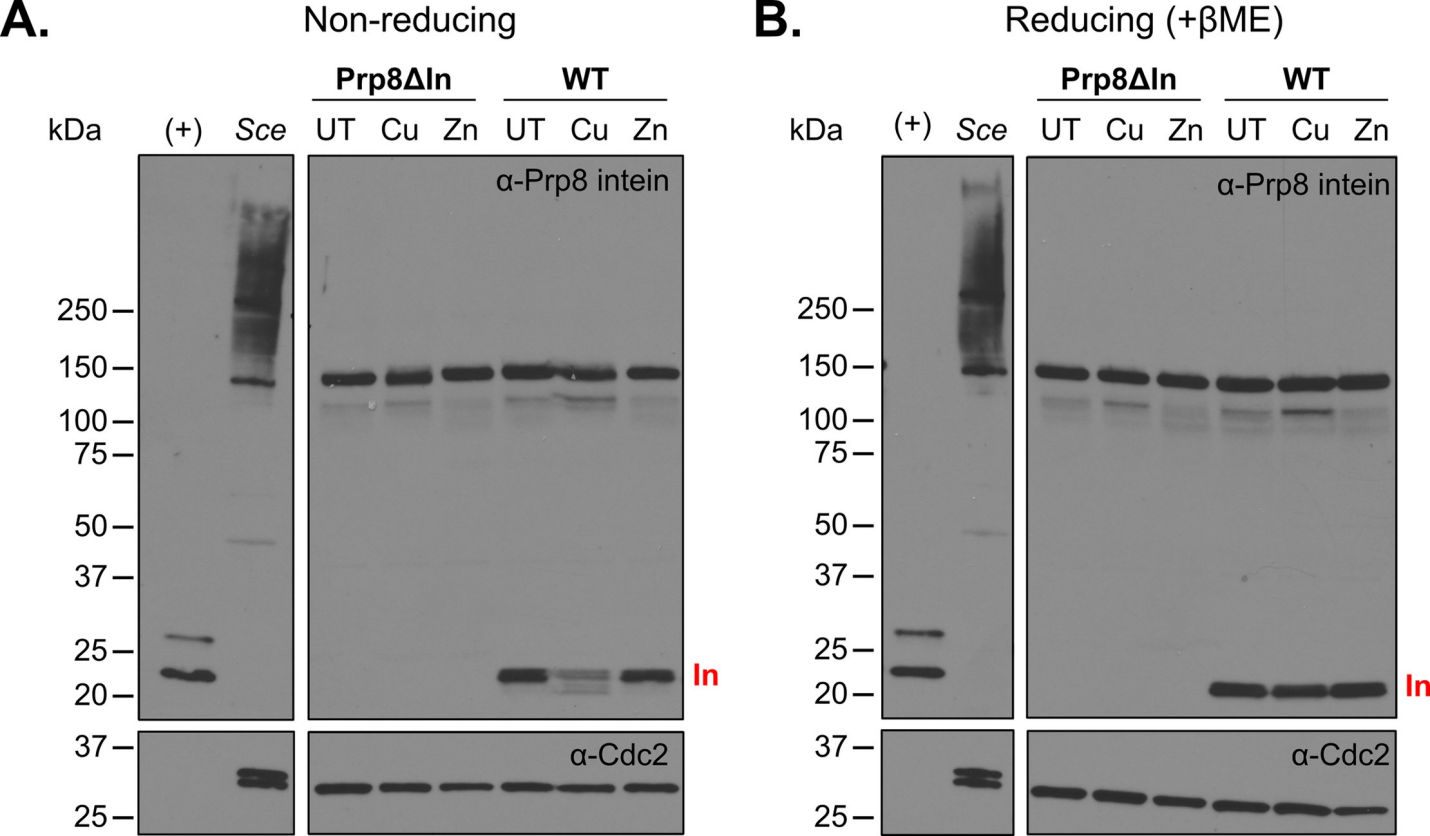
Native protein splicing in *Cne* is inhibited by copper. (A) Copper treatment shows decreased intein (In) levels under nonreducing conditions. *Cne* (WT) and an inteinless strain (Prp8ΔIn) were grown in defined media supplemented with 1 mM CuSO_4_ or 1 mM ZnSO_4_ for 3 h. Top blot is probed with Prp8 intein antibody, and the bottom blot is probed with Cdc2 antibody as a loading control. Intein bands at approximately 20 kDa often appear as doublets because of different oxidation states of cysteines. Bands migrating around 150 kDa that appear in the Prp8ΔIn and WT strain have not been identified, whereas bands from *Sce* lysate may be related to the Vma1 intein. Lanes are as follows: (+), 2.5 ng of overexpressed pET47b *Cne* Prp8 intein in *E*. *coli* lysate; *Sce*, 10 μg of soluble *S*. *cerevisiae* extract; UT, unsupplemented SC media; Cu, SC media + 1 mM CuSO_4_; Zn, SC media + 1 mM ZnSO_4_. All *Cne* lysates contain 20 μg of soluble protein. (B) Reducing conditions alleviate splicing inhibition in vitro. Blots are as in panel A but treated with βME in the loading dye. The reducing conditions in the lysate are sufficient to cause splicing in vitro and the intein band reappears. βME, β-mercaptoethanol; Cdc2, cyclin-dependent kinase 1; *Cne*, *C*. *neoformans*; Prp8, pre-mRNA processing factor 8; SC, synthetic complete; *Sce*, *Saccharomyces cerevisiae*; UT, untreated; WT, wild type.

To investigate whether metal stress affects protein splicing in vivo, in *Cne*, Western blotting was employed to probe for intein using a Prp8 intein antibody (Fig 6). The level of excised intein is a read-out for inhibition, because protein splicing is the most plausible pathway to yield free intein. We first determined that the intein antibody can detect the Prp8 intein, because it reacts with overexpressed *Cne* Prp8 intein at the expected size of approximately 20 kDa (Fig 6A and 6B, [+] lane). Moreover, the intein antibody does not detect any bands close to 20 kDa in an inteinless strain of *Cne* that we constructed (Fig 6A and 6B, Prp8ΔIn). Upon metal treatment, blots consistently revealed that incubation with CuSO_4_ (1 mM) caused a marked decrease in the amount of free intein (Figs 4C, 5A and 6A, Cu). Compared with unsupplemented media, copper treatment showed up to 50% reduction of free intein in *Cne*, correlating with the expected protein splicing inhibition. Importantly, this inhibition was relieved in vitro by addition of reducing agent β-mercaptoethanol to the treated lysate (Fig 6B), agreeing well with our MIG data that showed reversible cysteine modifications after copper incubation (Fig 5A). The ZnSO_4_ treated cultures show only a minimal decrease in free intein, possibly because the zinc, which we showed to be reversibly bound (Fig 5B), dissociates from the intein during lysate preparation. The intein antibody was unable to detect Prp8 precursor, perhaps because at a size of >290 kDa, the protein transfers poorly to the membrane. Regardless, these results indicate that Prp8 intein splicing is regulated in vivo, with implications for biological relevance to intron splicing.

### A precursor model of Prp8 relates intein retention to spliceosome function

**Fig 7:**
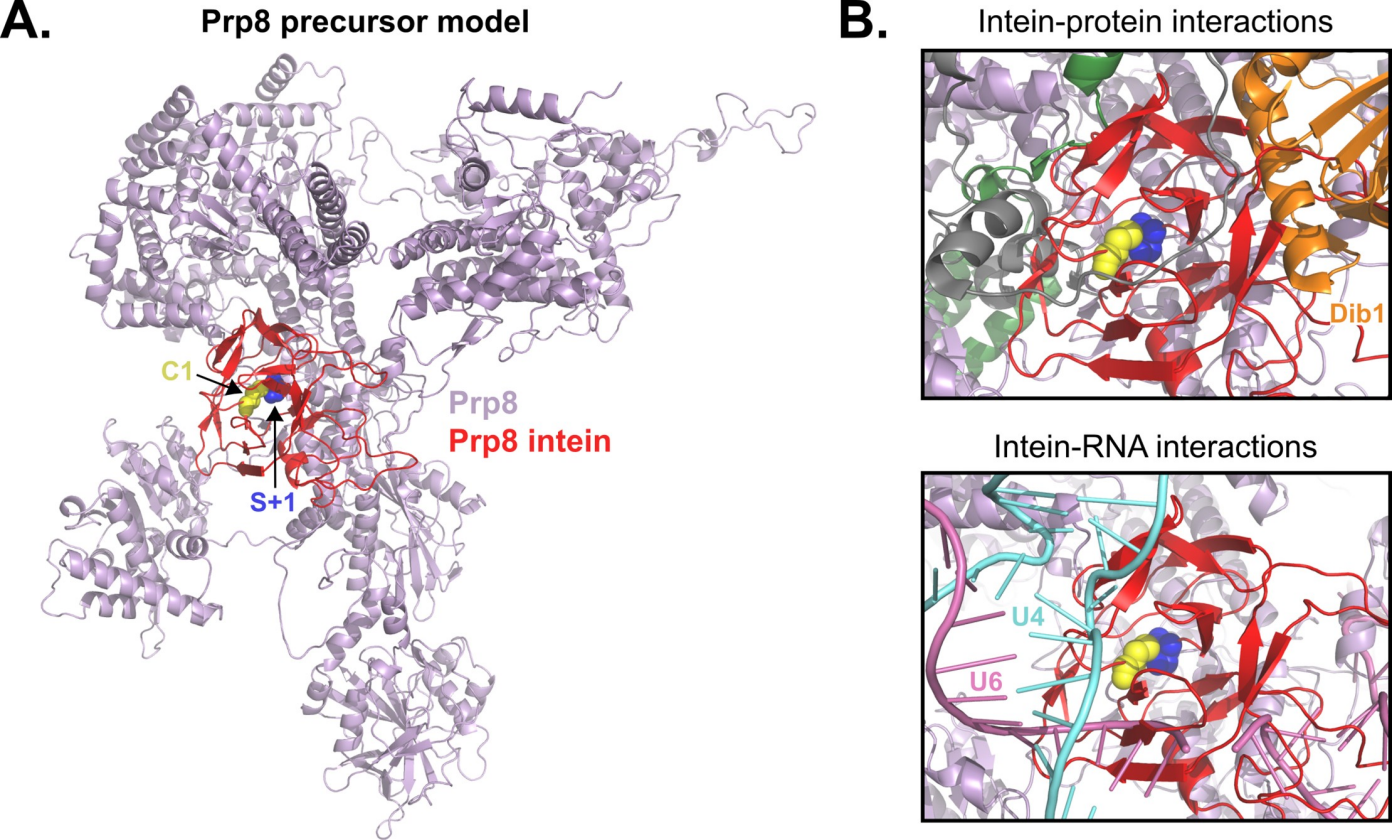
Modeling the *Cne* Prp8 intein into Prp8 and docking into the spliceosomal U4/U6.U5 tri-snRNP reveals unfavorable interactions. (A) *Cne* Prp8 intein in Prp8 exteins. The *Cne* Prp8 intein structure (red) was modeled into a structure of *S*. *cerevisiae* (PDB 5GAN, chain A) Prp8 exteins (lavender). The S+1 is shown as blue spheres to indicate the site where a peptide bond was broken to insert the intein. The C1 is shown as yellow spheres and specifies start of the intein. The Prp8 intein localizes to a crowded site of the Prp8 structure, in a region that is highly conserved and functionally important. (B) The *Cne* Prp8 intein in the spliceosomal U4/U6.U5 tri-snRNP. The intein-containing Prp8 was overlaid into the tri-snRNP subunit structure (PDB 5GAN). This revealed a clash of the intein with spliceosomal protein Dib1 (top panel, orange). There are also possible clashes with the intein and U4 snRNA and U6 snRNA (bottom panel, teal and pink, respectively). *Cne*, *C*. *neoformans*; PDB, Protein Data Bank; Prp8, pre-mRNA processing factor 8; snRNA, small nuclear RNA; tri-snRNP, triple small nuclear ribonucleoprotein.

Finally, we wished to ask how protein splicing inhibition might affect both Prp8 and the spliceosome. Therefore, we docked the intein into a known Prp8 structure and generated a precursor model, in which the intein is still covalently connected to the exteins. In this model, the bonds flanking the intein were broken at site a in Prp8 from the spliceosomal U4/U6.U5 triple small nuclear ribonucleoprotein (tri-snRNP) solved from *Sce* (Fig 7A, PDB 5GAN, chain A) [12,61]. The *Cne* Prp8 intein structure was computationally inserted using an energy optimization protocol, allowing insight into how intein presence might affect Prp8 and spliceosome assembly or activation.

Upon docking, it appears that the *Cne* Prp8 intein is accommodated in the Prp8 protein, albeit in a crowded area that would otherwise be occupied by helical folds (Fig 7A). The insertion site is in the 1585 loop, 1 of 3 structural motifs responsible for directly facilitating both steps of intron splicing [62]. Presence of the intein likely interrupts Prp8 function; given the importance of this location, the supporting contacts Prp8 makes within the spliceosome, and the RNA splicing defects in Prp8 mutants [1,2] (see Discussion). Mapping the other insertion sites onto another Prp8 structure from *Sce* also reveals that their presence would presumably disrupt Prp8 function, because they too cluster around the active site (S10 Fig, PDB 5GMK, chain A) [10].

We next overlaid the Prp8 intein-containing precursor in the tri-snRNP from *Sce* (Fig 7B, PDB 5GAN) [61]. It appears that the intein now occupies a cramped area of the spliceosome (S11 Fig). There are a few crucial components, both protein and RNA, in the vicinity of the intein. For example, one essential splicing protein, the U5 component Dib1, is located in the same 3D space as the intein (Fig 7B, top) [63]. Furthermore, there are important RNAs in the area of the intein (Fig 7B, bottom). These include the U4 and U6 snRNAs, which are central to spliceosome activation and RNA splicing catalysis [62]. Generally, the information gleaned from our precursor model suggests that the tri-snRNP subunit of the spliceosome would be disturbed by intein retention before spliceosomes are even fully formed, which would lead to inhibition of RNA splicing.

## Discussion

Here, we have shown that Prp8 inteins are widely distributed across eukaryotes and have invaded the Prp8 protein repeatedly and independently (Fig 1A and 1B), suggestive of potential adaptation that provides an advantage to the host. The crystal structure of the *Cne* Prp8 intein showed similarities to the metazoan Hedgehog protein and has facilitated studies of function as well as provided a basis for molecular modeling (Figs 2, 3 and 7). Initial studies demonstrated that some environmental stressors that are prevalent in infected macrophages are capable of modulating protein splicing of the *Cne* Prp8 intein, both in vitro and in vivo (Figs 4C and 6). Specifically, copper and zinc are potent inhibitors of protein splicing, with each metal interacting with the intein in distinct ways (Fig 5). Copper likely hinders protein splicing by cysteine oxidation and zinc inhibits by tenacious binding to the intein (Fig 5; S9 Fig). This work supports a growing theme in intein research that underscores the reactivity of catalytic cysteines [21–23,59,60]. We propose that the *Cne* Prp8 intein, at the nexus of protein and RNA splicing, may sense metals to pause Prp8 function during stressful conditions. This is reinforced by an intein-containing Prp8 precursor model, which suggests that protein splicing inhibition would interfere with Prp8 activity and disrupt full spliceosome assembly (Fig 7; S11 Fig).

### Prp8 is an intein sink with functional implications

We demonstrated a broad distribution of Prp8 inteins with multiple insertion sites (Fig 1A and 1B), a pattern noted by others as well [27,31,33]. Our data support the notion that Prp8 was invaded repeatedly, including at least twice at site a (Fig 1A, a1 and a2; S1A Fig). Previously, a limited number of site a inteins was analyzed and shown to be highly similar to each other [35]. We further discovered a novel insertion in a social amoeba, site g, bringing the total number of known insertion sites in Prp8 to 7 (Fig 1C; S3 Fig). This preponderance suggests Prp8 inteins have been retained over evolutionary time with functional implications. Similar trends were formerly reported with the mycobacterial iron-sulfur cluster assembly protein SufB, which has 3 distinct insertion sites, and the mycobacteriophage terminase TerL, which has at least 5 intein insertion sites [20,21,26]. Such bioinformatics observations have led to fruitful research on intein function, which is now beginning to show that inteins can be tuned to respond to environmental cues [19,21,23,24]. A striking example is a mycobacterial intein in DnaB helicase, located in the P loop of the ATPase domain [64], which is sensitive to ROS both in vitro and in vivo [59].

### Structural insights into the Prp8 intein

Here, we present the second structure of a eukaryotic intein, and the sole structure of a eukaryotic intein in an essential protein (Fig 2A). The *Cne* Prp8 intein structure provides insight into the similarity of inteins in eukaryotes (Fig 3A), suggesting that they likely evolved from a common ancestor. The *Cne* Prp8 intein also has a comparable structure to the C terminus of a Hedgehog protein (Fig 3C), which executes a cleavage and ligation reaction to cholesterol also by utilizing a cysteine [44]. These results suggest that eukaryotic inteins and Hedgehog proteins might be ancestrally related, but why inteins do not exist in metazoan genomes is a puzzle yet to be explained.

Around a dozen intein structures have been solved so far, comprising of mainly bacterial and archaeal inteins [65]. These have proven useful for studying inteins as novel drug targets [52]. As inteins often invade essential proteins in pathogens, inhibiting them from splicing out is an attractive option for developing new antimicrobials [52,66,67]. Progress toward this goal has been made in prokaryotes using the mycobacterial RecA recombinase intein. A co-crystal of the RecA intein and the antineoplastic compound, cisplatin, helped resolve the mechanism of protein splicing inhibition [52]. This showed that the platinum ions of cisplatin bind to the RecA intein at its 2 catalytic cysteines, C1 and C+1. Concurrent work studying cisplatin and the Prp8 intein also demonstrated effective splicing inhibition, both in vitro and in a mouse model, although the mechanism is different than the RecA intein [68]. Solving the *Cne* Prp8 intein structure, along with the observed metal inhibition, provides impetus for advancing these studies in an essential protein in a eukaryote, at an opportune time given that the antifungal pipeline is drying up [69].

### *Cne* Prp8 intein is responsive to metals in vitro and in vivo with biological ramifications

The *Cne* Prp8 intein was studied here using MIG, a GFP splicing reporter, given that full-length Prp8 could not be expressed well in *E*. *coli*. Studies have shown that intein splicing with surrogate exteins can be less effective than with native exteins [70,71]. However, we found that the wild-type *Cne* Prp8 intein spliced well in the MIG reporter. This illuminates how important extein context is in either constraining or allowing splicing of the *Cne* Prp8 intein. Previous studies placed the *Cne* Prp8 intein into non-native contexts and showed splicing, but contrary to our work, there was almost complete inactivation by the A-1V mutation [48]. This earlier work only used 1 to 2 flanking extein residues, whereas MIG contains 5, suggesting that more native context allows greater tolerance to sequence variation. Such discrepancies do raise the important point that the extein context of an intein is extremely important when asking questions about splicing.

Here, MIG Prp8 was useful in a screen for identifying the divalent metals copper and zinc as potential regulatory signals for protein splicing. Given that the intein-containing organism is the pathogenic fungus *Cne*, we wondered whether metal-based inhibition contributes to a stress response during infection. Pathogenic microbes occupy niches that expose them to the opposing toxicities of metal ion excess and deprivation [72]. *Cne* employs extensive strategies to control metal ion concentrations, including responsive transcription factors, transporters, importers, and exporters [73]. Many of these are utilized during the oxidative burst of the phagolysosome when the fungus is exposed to acute metal stress [73]. Levels of copper can reach up to several hundred micromolar, while zinc concentrations are initially high, but drop with ongoing infection [72,74,75].

We speculate that the *Cne* Prp8 intein might provide cryptococci an additional means to sense metals during infection. The sensing “machinery” of the *Cne* Prp8 intein is its catalytic C1 and N171 (Fig 5). Cysteines are reactive amino acids that endow proteins with catalytic activity, redox chemistry, and metal binding capacity, whereas asparagines can be both catalytic and metal-coordinating residues [58]. A pause in protein splicing may be useful for overcoming toxic levels of copper or zinc (Figs 4C, 5 and 6). Copper generates destructive ROS intermediates and can displace iron from iron-sulfur clusters [74], whereas both copper and zinc can dislodge divalent metals from other metalloprotein complexes. Like other stressors known to inhibit RNA splicing, copper and zinc would act post-translationally to block Prp8 intein splicing and inhibit spliceosome function until levels of the metals are diminished by scavenger proteins or metal transporters [73]. Indeed, we provide the first evidence here that Prp8 intein splicing in *Cne* can be modulated under metal stress (Fig 6).

Post-translational programs that regulate expression of intron-containing transcripts in response to environmental cues have been described in the budding yeast, *Sce* [76] and in *Cne* [77]. Work done on alternative splicing in *Cne* supports pausing of spliceosome function [77]. This fungus is intron dense, with over 40,000 introns in its genome, and abundant alternative splicing has been observed [29]. Intriguingly, the most common type of aberrant splicing is intron retention [77]. Intron retention has even been shown to play a role in virulence and is regulated by environmental conditions [77]. If intron retention is an adaptive mechanism for *Cne* to finely tune expression levels in adverse environments, then inhibiting Prp8 intein splicing is a possible means of controlling that intron retention.

### Protein splicing inhibition and its implications for RNA splicing

We turned to molecular modeling of structures to help predict in vivo effects of intein splicing inhibition. The intein-containing Prp8 precursor model generated from a solved *Sce* U4/U6.U5 tri-snRNP revealed a snug accommodation of the intein in a highly conserved region of Prp8 (Fig 7A, PDB 5GAN, chain A) [61]. This insertion (site a) is in a linker located between the thumb domain and the endonuclease-like domain of the reverse transcriptase (Fig 1B; S10 Fig). This highly conserved region of Prp8 (55%–87% identity over 113 residues) contains the 1585 loop and its longer version, the α-finger [62]. These structural motifs are involved in coordinating the RNA-mediated catalysis that leads to eventual intron removal [1,62], at the core of the protein and at the catalytic center of the spliceosome.

Although Prp8 likely cannot perform its critical functions with the intein present, the structural tolerance of a flexible linker domain may allow for proper folding of the intein, as well as that of Prp8. In the longer term, this flexibility gives the intein freedom to adapt to its surroundings, supporting some degree of Prp8 function in a precursor state. Mini-inteins, such as the one present in *Cne* Prp8, are not mobile and may therefore be under more selective pressure to adapt to their exteins. This is in line with work that shows partial activity of the RadA precursor with its mini-intein intact [19].

If the *Cne* Prp8 intein remains unspliced in the tri-snRNP, perhaps because of metal sensing and inhibition, it would almost certainly be disruptive. In the subunit modeled here, the intein would overlap with the Dib1 protein (Fig 7B, top) [61], which is an essential spliceosome component. Dib1 is a small, 16.8 kDa protein well-conserved from yeast to humans and is postulated to play a central role in preventing premature spliceosome activation [63]. If Dib1 is unable to localize to its cognate site, this would likely be detrimental to spliceosomal function [63]. Furthermore, certain RNAs, such as U4 and U6, thread close to the intein (Fig 7B, bottom). Dislodging these critical snRNAs would almost certainly disrupt intron splicing.

If the *Cne* Prp8 intein were to sense a stressor, such as metals, and stay lodged in Prp8, then Prp8 precursor would undoubtedly affect spliceosome assembly and possibly activation (Fig 7; S11 Fig). Such spliceosome assembly defects may lead to pre-mRNA accumulation, as even point mutations in Prp8 are known to do [2]. Thus, this work proposes that the *Cne* Prp8 intein is subject to modifications that influence protein splicing and thereby Prp8 function, with implications for spliceosome activity.

## Materials and methods

### Bioinformatic and phylogenetic analyses

The Prp8 intein sequences used to build the phylogenetic trees in Fig 1A and S1 Fig and the Prp8 extein sequences used to build the phylogenetic trees in S4 Fig were accessed from Green and colleagues, 2018 [27]. For comparative and phylogenetic analyses, amino acid sequences of inteins were manually trimmed to the splicing blocks (A, B, F, and G). All multiple sequence alignments of the amino acid sequences were performed using ClustalOmega with default parameters [78] and edited manually (Fig 1; S1, S2 and S4 Figs). Where alignments are shown shaded, black represents an identical amino acid, dark gray is a conserved amino acid, whereby the same amino acid is at the same position in a majority of the sequences, and light gray is a similar amino acid, defined as a semiconserved amino acid substitution from the same class. Phylogenetic analysis was performed using the neighbor-joining (NJ) method in the MEGA7 program [79]. Statistical support for the NJ tree was evaluated by interior-branch test (number of replications, 1,000) [80]. The sequence logo for Block B was generated based on the multiple sequence alignment using WebLogo3 [81] (http://weblogo.threeplusone.com). The 7 Prp8 intein insertions were mapped onto a model of a *Sce* Prp8 (S10 Fig, PDB 5GMK). All intein, Prp8, and spliceosome structures were viewed, edited, or aligned using PyMol 1.3 (http://pymol.org). The 3D BLAST protein structure search was performed by BioXGEM with default parameters (http://3d-blast.life.nctu.edu.tw).

### Bacterial strains and growth conditions

All strains used in the present study can be found in S1 Table. *E*. *coli* DH5α, MG1655(DE3), and BL21(DE3) were grown in Luria Broth (LB), unless otherwise indicated, with aeration at 250 rpm. Media contained kanamycin (50 μg/mL) or chloramphenicol (25 μg/mL) where appropriate. Plasmids were transformed into cells by electroporation using a Bio-Rad Gene Pulser (Hercules, CA) and recovered for 1 h at 37°C in SOC medium (0.5% yeast extract, 2% tryptone, 10 mM NaCl, 2.5 mM KCl, 10 mM MgCl_2_, 10 mM MgSO_4_, and 20 mM glucose). Transformants were selected by plating on LB agar with the appropriate antibiotic and incubated at 37°C overnight.

### Construction of plasmids

All plasmids used in the present study can be found in S2 Table and all oligonucleotides, synthesized by Integrated DNA Technologies (IDT, Coralville, IA), are in S3 Table. Plasmid DNA was prepared using EZNA Plasmid Mini Kit (Omega, Norcross, GA). DNA was visualized in 1% agarose gels using EZ-Vision DNA Dye (Amresco, Radnor, PA). PCR fragments were amplified using CloneAmp HiFi PCR Premix (Clontech, Mountain View, CA) from genomic DNA of *Cne* var. *grubii* H99 or *C*. *gattii* NIH444 (Dr. Sudha Chaturvedi, New York State Department of Health), *A*. *fumigatus* AF293 (Dr. Robert J. Cramer, Darmouth College), *B*. *dendrobatitidis* JEL423 (Dr. Timothy James, University of Michigan), or *H*. *capsulatum* G186A (Dr. Chad Rappleye, Ohio State University). For insertion into the MIG construct, the inserts included 5 native N- and C-extein residues flanking the intein. For insertion into the overexpression vector, pET47b, the intein alone with 3 native N exteins was PCR amplified. Digested backbone was gel purified using Zymoclean Gel DNA Recovery Kit (Zymo Research, Irvine, CA). Restriction enzymes (NEB, Ipswitch, MA), T4 ligase (NEB, Ipswitch, MA), and In-Fusion HD Cloning Plus Kit (Clontech, Mountain View, CA) were all used per manufacturer protocol. Mutagenesis was performed using the QuikChange Lightning Site-Directed Mutagenesis Kit (Agilent, Santa Clara, CA) for single amino acid mutations or the QuikChange Lightning Multi Site-Directed Mutagenesis Kit (Agilent, Santa Clara, CA) for multiple amino acid mutations. For the A-1V mutation, primers were designed to randomly mutate the A-1 to all other possible codons using a degenerate primer with NNS at the mutated location. All clones were verified by sequencing (EtonBio, Union, NJ).

### MIG splicing assays

MIG Prp8 (WT, A-1V, and derived C61 mutants) was transformed by electroporation into MG1655(DE3). The cells were subcultured 1:100 from an overnight culture into fresh LB medium and grown at 37°C with 250 rpm shaking to an OD_600_ of 0.5. Cells were induced with 0.5 mM IPTG for 1 h at 30°C and pelleted by spinning for 10 min at 4,000 rpm. The pellets were lysed immediately using tip sonication (20 s on/30 s off at 30% amplitude for 1 min total) in 50 mM Tris (pH 8.0) and 10% glycerol or stored at −80°C until lysis. For any ROS/RNS or metal treatment, the indicated compound was added to cells at the desired concentration prior to incubation at 30°C for the specified time. For the assays to determine mechanism of inhibition, MIG Prp8 A-1V lysate was split, and half was left untreated, whereas the other was preincubated with 1 mM CuSO_4_ or ZnSO_4_. After 2 h, each lysate (with metal or untreated) was split in half and either treated with EDTA to a final concentration of 10 mM or with TCEP to a final concentration of 40 mM. Aliquots of the EDTA/TCEP-treated lysates were then collected immediately at t0, 2 h, and 22 h postincubation with EDTA/TCEP. Because EDTA/TCEP is added after an initial 2 h incubation, these new t0, 2 h, and 22 h samples actually represent 2 h, 4 h, and 24 h since start of the assay. Upon completion of the assay or time point, the lysate was frozen at −80°C. To visualize MIG splicing assay results, samples were separated under nonreducing conditions on Novex WedgeWell 12% Tris-Glycine gels (Invitrogen, Carlsbad, CA) using loading dye lacking β-mercaptoethanol and visualized using a Typhoon 9400 scanner (GE Healthcare, Chicago, IL) with excitation at 488 nm and emission at 526 nm. Quantitation and analysis were done using ImageJ and GraphPad Prism (version 7.02).

### Prp8 intein purification

For isothermal titration calorimetry and mass spectrometry, the *Cne* Prp8 intein from *Cne* var. *grubii* H99 was amplified with 3 native N-extein residues (EKA) and cloned into pET47b in front of an N-terminal His_6_-tag and an HRV 3C protease site. For crystallization, the *Cne* Prp8 intein with 2 native N-extein residues (KA), and the native C-extein S+1 was amplified and cloned into pET28a with a C-terminal His_6_-tag using a megaprimer approach as described previously by Li and colleagues [82].

The pET47b (or pET28a) *Cne* Prp8 intein construct was transformed by electroporation into BL21(DE3) cells. The cells were subcultured 1:100 from an overnight culture into fresh LB medium and grown to an OD_600_ of 0.6. Cells were induced with 0.5 mM IPTG and grown with shaking at 250 rpm overnight at 16°C. The following morning, cells were harvested by centrifugation at 4,000 rpm for 10 min. Pellets were frozen at −80°C until ready for lysis. Tip sonication was performed (30 s on/59 s off at 30% amplitude for 4 min total) in buffer containing 20 mM Tris (pH 7.8), 500 mM NaCl, 25 mM imidazole, and 5% glycerol. Whole cell lysate was centrifuged at 20,000*g* for 20 min to separate the soluble fraction, which was loaded onto a nickel affinity column equilibrated with the lysis buffer. Washes were carried out using buffer containing 20 mM Tris (pH 7.8), 500 mM NaCl, 75 mM imidazole, and 5% glycerol and elution buffer with 20 mM Tris (pH 7.8), 500 mM NaCl, 250 mM imidazole, and 5% glycerol. Purified fractions of the *Cne* Prp8 intein were checked by separation on SDS-PAGE, and the cleanest elution samples were pooled. For the pET47b construct, the His_6_-tag on the *Cne* Prp8 intein was removed through digestion with HRV 3C protease according to the manufacturer’s protocol (Thermo Fisher, Waltham, MA). The cleaved *Cne* Prp8 intein reaction was passed back over a nickel affinity column, and the flow-through was collected to ensure no His_6_-tagged *Cne* Prp8 intein or HRV 3C protease was in the sample. For analysis by ITC, the flow-through intein was exchanged into 50 mM sodium acetate (pH 7.0), 100 mM NaCl using a HiPrep 26/10 desalting column or a dialysis cassette. For mass spectrometry, the flow-through intein was used directly for metal treatments and then further purified by liquid chromatography (LC) prior to spraying on the instrument. For the pET28a construct, the imidazole-eluted fractions were concentrated and subjected to size exclusion chromatography by a gel filtrations 16/60 Superdex column (GE Healthcare, Chicago, IL). For crystallization, the purified *Cne* Prp8 intein was concentrated to 9.5 mg/mL in a buffer composed of 25 mM HEPES (pH 7.5) and 150 mM NaCl.

### Mass spectrometry of Prp8 intein

Purified *Cne* Prp8 intein was reduced with 40 mM TCEP and exchanged into deoxygenated exchange buffer (20 mM Tris [pH 7.5], 200 mM NaCl) using 7K MWCO Zeba spin desalting columns (Thermo Fisher, Waltham, MA) to remove TCEP. The protein concentration was measured and then treated with 10X of CuSO_4_ and incubated at 30°C for 1 h. Following treatment, the purified intein was denatured with 6 M urea at 37°C for 30 min. The urea concentration was diluted down to less than 0.8 M with 50 mM Tris (pH 7.6) and 1 mM CaCl_2_. Trypsin digest of the intein was performed by adding activated trypsin (Promega, Madison, WI) to a final ratio of 1:20 and incubating overnight at 37°C. The oxidation of *Cne* Prp8 intein cysteines after treatment was analyzed by multiple reaction monitoring-initiated detection and sequencing (MIDAS) as described by Unwin and colleagues [83]. The trypsin-digested mixture was acidified followed by LC-MS/MS analysis. LC-MS/MS analysis was performed on a microflow LC-MS/MS system configured with a 3-pumping Micromass/Waters CapLC system with an autosampler, a stream select module configured for precolumn plus analytical capillary column, and a QTRAP 6500 (ABSCIEX) mass spectrometer fitted with Turbo V microflow source, operated under Analyst 1.63 control. Injected samples were first trapped and desalted isocratically on an LC-Packings PepMa C18 μ-Precolum Cartridge (5 μm, 500 μm ID × 20 mm; Dionex, Sunnyvale, CA) for 7 min with 0.1% formic acid delivered by the auxiliary pump at 40 μL/min after which the peptides were eluted from the precolumn and separated on an analytical C18 capillary column (15 cm × 500 μm ID, packed with 5 μm, Jupiter 300 C18 particles, Phenomenex, CA) connected inline to the mass spectrometer at μL/min using a 50 min gradient of 5% to 80% acetonitrile in 0.1% formic acid. The oxidized peptide identification was conducted through multiple reaction monitoring (MRM) triggered enhanced product ion (EPI) scan using information dependent acquisition (IDA). The utilization of chromatographic separation, MRM transitions, and EPI scan allows accurate peptide identification and confirmation. The 2 MRM transitions including m/z 404.19 > 532.22 and m/z 786.04 > 895.41 for C[Oxi]LQNGTR.+2b5 and THEGLEDLVC[Oxi]THNHILSMYK.+3b8 were used to trigger the EPI experiment, respectively. The instrument was operated in a positive ion mode with a Turbo V ion drive electrospray source. The parameters for the operation were as follows: curtain gas, 20 psi; heated nebulizer temperature 180°C, ion spray voltage, 5,500 V; gas1, 18 psi; gas 2, 15 psi, declustering potential, 65 V, EP, 10 V and CAD gas, high.

### ITC of Prp8 intein

ITC measurements were carried out on a TA Instruments Nano ITC (TA Instruments, Inc., New Castle, DE). Aqueous solutions of metal titrants (CuSO_4_ or ZnSO_4_) were prepared to be 0.3- to 30-fold higher than the concentration of the *Cne* Prp8 intein, in the range of 0.05 to 5.0 mM. The titrant and wild-type *Cne* Prp8 intein were degassed before each titration. The purified *Cne* Prp8 intein was concentrated from 10 μM to 16 μM in 300 μL and were placed in a 2.5 mL reaction cell, and the reference cell was filled with 300 μL deionized water. All titrations were carried out at 37°C. After baseline equilibration, successive injections of an indicated titrant were made into the reaction cell in 2.5 μL increments at 400 s intervals with stirring at 250 to 350 rpm to ensure an equilibrium was achieved for a return to baseline. The resulting heats of reaction were measured over 20 consecutive injections. Optimization of buffer was required for purified *Cne* Prp8 intein and found to be stable over long periods for ITC data collection only in 50 mM sodium acetate (pH 7.0), 100 mM NaCl, 10 mM TCEP. Buffer control experiments (50 mM sodium acetate [pH 7.0], 100 mM NaCl, ± 10 mM TCEP) to determine the heats of titrant dilution were carried out by making identical injections in the absence of the *Cne* Prp8 intein. The net reaction heat was obtained by subtracting the heats of dilution from the corresponding total heat of reaction. The titration data were deconvoluted based on best-fit binding models containing either 1 or 3 sets of interacting binding sites, using a nonlinear least-square algorithm through the NanoAnalyze software. The binding enthalpy change (ΔH), dissociation constant (K_d_), and binding stoichiometry (n) were permitted to vary during the least-square minimization process and taken as best-fit values.

### Crystallization, structure determination, and refinement of Prp8 intein

Initial crystallization conditions were obtained by screening the Hampton crystallization screens (I, II, and Research Index HT), using the hanging-drop vapor diffusion method. Upon optimization, large crystals were grown by mixing 1 μL of *Cne* Prp8 intein and 1 μL of reservoir solution containing 22% to 28% PEG4000, 0.1 M sodium acetate (pH 4.2), 0.2 M ammonium acetate. The *Cne* Prp8 intein crystallizes in space group *P1* with 6 intein molecules per asymmetric unit (S4A Table). The crystals of the *Cne* Prp8 intein-Zn^2+^ complex were obtained by soaking native crystals in mother liquor supplemented with 4 mM zinc acetate. Prior to data collection, all crystals were transferred to a cryo-protectant solution containing crystallization buffer supplemented with 20% glycerol without (native) or with 4 mM zinc acetate (Prp8-Zn^2+^ complex). The crystals were flash-cooled directly in liquid nitrogen. Diffraction data for the native and the Prp8-Zn^2+^ complex crystals were collected at 100 K using a Pilatus detector at the BL9-2 beamline of the Stanford Synchrotron Radiation Laboratory (native) and using an ADSC HF-4M detector at the 19-ID NYX beamline of the National Synchrotron Light Source II (complex), respectively. Data were processed, scaled, and reduced using the programs HKL2000 [84] and PHENIX suite [85]. The structure of the *Cne* Prp8 intein was determined by molecular replacement, with the crystal structure of the *C*. *gattii* Prp8 intein [68] as a search model. The Prp8-Zn^2+^ complex structure was determined by molecular replacement, with the refined structure of the *Cne* Prp8 intein as a search model. The molecular replacement was carried out using the PHENIX program suite. Structure refinement was carried out using the PHENIX program suite and monitored using Coot [86] (S4B Table).

### Generation of an inteinless *Cne* (Prp8ΔIn)

The synthetic inteinless *PRP8* gene (designated *PRP8ΔIn*) was cloned into the *Cne* Safe Haven vector pSDMA25 [87]. Plasmid pSDMA25-Prp8ΔIn was linearized with PacI (NEB, Ipswitch, MA) and used for biolistic transformation of wild-type *Cne* H99. After selection on YPD agar plates containing nourseothricin (NAT), insertion at the Safe Haven site was validated by multiplex PCR. Both the intact wild-type locus of *PRP8* as well as the *PRP8ΔIn* locus within the Safe Haven site were verified by PCR. To construct a knock-out of the wild-type *PRP8* gene, a hygromycin (*HYG*) cassette flanked by 1 kb of *PRP8* sequence was used to transform *Cne* harboring *PRP8ΔIn* at the Safe Haven site. *HYG* was used as the selection marker. Proper deletion of wild-type *PRP8* and retention of *PRP8ΔIn* at the Safe Haven site, generating the strain Prp8ΔIn, was again validated by PCR amplification as well as by Southern blotting.

### *Cne* culture and Western blot

*Cne* H99 derivatives and *Sce* S288C were grown in defined synthetic complete media (SC) after diluting overnight cultures to an OD_600_ of 0.2 in fresh SC. After 3 h at 30°C, 200 rpm, cells were supplemented with 1 mM CuSO_4_ or ZnSO_4_. Cells were incubated an additional 3 h at 30°C, 200 rpm, harvested, washed with 3 × 5 mL PBS and resuspended in 0.6 mL lysis buffer (25 mM Tris [pH 7.4], 140 mM NaCl, 1 mM EDTA, 1% Triton X-100, 1 mM PMSF, and 1× fungal specific protease inhibitor [Sigma Aldrich, St. Louis, MO] and lysed with acid-washed glass beads in a Bead Ruptor 12 (Omni International, Keenesaw, GA). Cell debris was centrifuged for 10 min at 16,000*g* and soluble extracts were mixed with 4x Laemmli Sample Buffer (Bio-Rad, Hercules, CA) either with or without added β-mercaptoethanol. Samples were then boiled for 10 min at 90°C and 20 μg total protein loaded on a 4% to 20% Criterion TGX gel (Bio-Rad, Hercules, CA), electrophoresed at 150 V in SDS running buffer for 1 h, then transferred onto a nitrocellulose membrane using a TransBlot Turbo system (Bio-Rad, Hercules, CA). Transfer quality was assessed by Ponceau-S stain. Membranes were washed 2 × 10 min in PBS with Tween-20 (PBST), then blocked for 1 h in 5% milk PBST. Membranes were washed again 2 × 5 min in PBST, then probed with primary anti-Prp8 intein (1:1,000) or anti-Cdc2 (1:5,000) in 2% milk PBST overnight at 4°C. Membranes were washed 3 × 15 min with PBST and then applied with HRP-conjugated secondary antibodies (GE Healthcare, Chicago, IL; rabbit or mouse at 1:10,000) in 2% milk PBST and incubated for 1 h. Blots were washed again 3 × 15 min with PBST, and then applied with SuperSignal West Femto (Thermo Scientific, Waltham, MA) chemiluminescent reagents, and images developed using X-ray film at different exposures.

### Precursor modeling

The Prp8 protein structure (chain A) in the 3.7 Å cryo-EM structure of the *Sce* spliceosomal tri-snRNP [61] (PDB 5GAN) was used as a structural template for constructing a homology model of the *Cne* Prp8 extein. A pair-wise Needleman-Wunsch [88] sequence alignment using EMBOSS Needle [89] shows 55.3% sequence identity and 68.3% sequence similarity between the full *Sce* and *Cne* extein sequences. The *Cne* Prp8 extein homology model was constructed using MODELLER [90] with the DOPE [91] and GA341 [92] energy functions to identify the best model. The 171-residue *Cne* intein sequence is an insert between residues 1530 and 1531 in the extein. The *Cne* Prp8 extein homology model was combined with the *Cne* Prp8 intein crystal structure to generate a model for the full *Cne* Prp8 precursor. The orientation of the intein with respect to the extein was manually adjusted using VMD software [93] to avoid any steric overlap and keep the intein and extein ends that are joined by peptide bonds sufficiently close together. This combined intein-extein structure was then used as a structural template to generate a continuous *Cne* Prp8 precursor homology model using MODELLER. Further minimization on this precursor homology model was performed using the program CHARMM, version c35b3 [94,95] with the CHARMM36 force field for proteins [96]. All atoms not within the precursor amino acid sequence range encompassing the intein and its neighboring extein regions (residues 1520–1720) were initially held fixed, and a low-temperature (150 K) optimization protocol was used to improve the homology model. This protocol included 5 iterations of the following steps: (a) 5,000 steps of Steepest Descent (SD) minimization followed by 5,000 steps of Adapted-Basis Newton-Raphson (ABNR) minimization, each with an energy change tolerance of 0.001 kcal/mol; (b) 1,000 steps of Langevin dynamics at a temperature of 150 K and a friction coefficient of 5.0 ps^−1^; (c) another 5,000 steps of SD and 5,000 steps of ABNR minimization. All nonhydrogen atoms were then restrained using harmonic restraints with a force constant of 1.0 kcal/mol/Å^2^. SHAKE constraints [97] were applied on all hydrogen atoms, and 5,000 steps of SD and 5,000 steps of ABNR minimization were performed to obtain the final *Cne* Prp8 precursor model.

## Supporting information

S1 Fig Distribution of Prp8 inteins. (A) A phylogenetic tree of Prp8 inteins was reconstructed based on an amino acid multiple sequence alignment of the splicing blocks (A, B, F, G) using the NJ algorithm and an interior-branch test with 1,000 replicates. Fifty representatives covering Prp8 intein diversity were selected, and the full name of each intein-containing organism is listed. Colored symbols represent the insertion site and correspond to colors in Fig 1A. Letters (a1, a2, b, c, d, e, f, g) represent each of the 7 unique insertion sites. (B) A phylogenetic tree of Prp8 inteins was reconstructed based on an amino acid multiple sequence alignment of the splicing blocks (A, B, F, G) using the ML method and evaluated with SH-aLRT. The substitution model, WAG+G+I, was selected using ProtTest 3 (https://github.com/ddarriba/prottest3). ML tree follows the same formatting as in panel A and shows similar architecture as NJ tree. Amoebo, Amoebozoa; Asco, Ascomycota; Basidio, Basidiomycota; Blasto, Blastocladiomycota; Choano, Choanoflagellida; Chloro Viridipl, Chlorophyta Viridiplantae; Chytridio, Chytridiomycota; ML, maximum likelihood; Mucoro, Mucoromycota; NJ, neighbor-joining; Opistho, Opisthokonta; Prp8, pre-mRNA processing factor 8; SH-aLRT, Shimodaira–Hasegawa nonparametric approximate likelihood-ratio test
(TIF)

S2 Fig Amino acid multiple sequence alignment of Prp8 inteins utilized for phylogenetic analysis. Comparative analysis of amino acid residues found in Blocks A, B, F, and G from the selected 50 representative Prp8 inteins, shown with abbreviated species names (full names in S1 Fig). Letters (a1, a2, b, c, d, e, f, g) represent each of the 7 unique insertion sites. Shading is as follows: black, identical amino acid; dark gray, conserved amino acid; light gray, similar amino acid substitution. Prp8, pre-mRNA processing factor 8
(TIF)

S3 Fig Novel Prp8 insertion site g. In the amoeba *Asu*, an intein was identified at a new site in Prp8, here termed g. This is the seventh site in which a Prp8 intein has been found. The full site g intein sequence is shown, plus 10 flanking N-extein (blue) and C-extein (green) amino acids. The *Asu* C1 (yellow) and terminal asparagine (red) are highlighted. Residue numbering corresponds to the *Asu* Prp8 exteins. Accession number: XP_0127532. Asu, *Acytostelium subglobosum*; Prp8, pre-mRNA processing factor 8
(TIF)

S4 Fig Conservation of Prp8 exteins. (A) A phylogenetic tree of Prp8 exteins corresponding to inteins (see S1 Fig) was reconstructed based on an amino acid multiple sequence alignment using the NJ algorithm and an interior-branch test with 1,000 replicates. Extreme conservation among Prp8 exteins is observed along with grouping by host organism phylogeny. Colored symbols represent the intein insertion site of the exteins and correspond to colors in Fig 1A. Letters (a1, a2, b, c, d, e, f, and g) represent each of the 7 unique insertion sites. Phylum abbreviations are listed in the S1 Fig legend. (B) A phylogenetic tree of Prp8 exteins was reconstructed based on an amino acid multiple sequence alignment of the splicing blocks (A, B, F, G) using the ML method and evaluated with SH-aLRT. The substitution model, LG+G, was selected using ProtTest 3 (https://github.com/ddarriba/prottest3). Tree follows the same formatting as in panel A. ML, maximum likelihood; NJ, neighbor-joining; Prp8, pre-mRNA processing factor 8; SH-aLRT, Shimodaira–Hasegawa nonparametric approximate likelihood-ratio test.
(TIF)

S5 Fig Overlays of the *Cne* Prp8 intein with other inteins. (A) Overlay of the *Sce* VMA1 intein and *Cne* Prp8 intein active sites. The *Sce* VMA1 intein (cyan, PDB 1GPP) was overlaid with the *Cne* Prp8 intein (red). The active site residues, crucial to protein splicing, are shown as sticks and labeled. A majority of these conserved residues overlap exactly, such as the catalytic C1, and the Block B TxxH motif. The *Sce* VMA1 intein uses an asparagine (N76) rather than threonine in the TxxH motif, but the positioning is similar to the threonine (T62) of the *Cne* Prp8 intein. The penultimate histidines (H170 and H453) are in comparable positions except for the side chains, whose chi angles are different by 45°. The *Sce* VMA1 intein was not solved with the terminal asparagine. (B) Structural comparison of bacterial *Mtu* RecA intein and fungal *Cne* Prp8 intein. Overlay of the *Mtu* RecA intein (brown, PDB 2IMZ), and the *Cne* Prp8 intein (red) reveals structural similarities in major intein features, such as the anti-parallel β-sheet folding, that contribute to the horseshoe shape. The Hint domain, comprised of splicing Blocks A, B, F, and G, are generally aligned between the 2 inteins. The structures deviate at sequences between Blocks B and F, where the *Cne* Prp8 intein encoded a linker or endonuclease domain. The 2 structures have an RMSD value of 2.22 Å. *Cne*, *C*. *neoformans*; *Mtu*, *Mycobacterium tuberculosis*; PDB, Protein Data Bank; Prp8, pre-mRNA processing factor 8; RMSD, Root-mean-square deviation; *Sce*, *Saccharomyces cerevisiae*
(TIF)

S6 Fig Splicing of Prp8-a inteins from other fungal pathogens in MIG. (A) Diverse Prp8 intein splicing patterns. Several Prp8 inteins from other fungal pathogens *Afu*, *Bde*, and *Hca* were cloned into MIG. Splicing was observed over time by the loss of precursor (P) and increase in LE, or simply by the presence of ligated exteins (for *Afu*). The gel shows that not all Prp8 inteins splice similarly, despite being placed in an identical extein context. (B) Precursor amounts vary greatly. A quantitation of precursor (P) at each time point shows that these Prp8 inteins are active but splice at variable rates. The *Afu* Prp8 intein is almost entirely spliced at the start of the assay (0 h), whereas *Bde* has 31% precursor at 0 h and *Hca* has 14% precursor at 0 h. Initial splicing rates were determined by calculating the loss of precursor over time (P_t0_−P_t1_/60 min) with standard error for MIG *Bde* Prp8 and MIG *Hca* Prp8, and are (5.9 ± 0.4) × 10^−2^% per min and (2.7 ± 0.9) × 10^−2^% per min, respectively. This suggests intein-mediated control of protein splicing. Data are representative of 3 biological replicates and mean standard deviations are shown. Trend lines are fit to show the decay curve. Data available in S1 Data. *Afu*, *Aspergillus fumigatus*; *Bde*, *Batrachochytrium dendrobatidis*; Hca, *Histoplasma capsulatum*; LE, ligated exteins; MIG, MBP-Intein-GFP; Prp8, pre-mRNA processing factor 8
(TIF)

S7 Fig MIG Prp8 A-1V copper inhibition and cysteine analysis. (A) Copper treatment causes inhibition. Induced MIG Prp8 A-1V cells were lysed and treated with 0 or 1 mM CuSO_4_. The lysates were incubated for the indicated time at 30°C and then frozen. Samples were separated on SDS-PAGE and scanned for GFP fluorescence. In the absence of copper, MIG Prp8 A-1V spliced well over 30 h, converting P into LE. There was little to no conversion of P to LE over time with copper addition. Quantitation is shown below in a stacked plot. Data are representative of 3 biological replicates and mean standard deviations are shown. Data available in S1 Data. (B) Relative position of 2 cysteines. There are only 2 cysteines present in the *Cne* Prp8 intein. Using the solved structure, a measurement of the distance between C1 and C61 (shown as sticks) was calculated to be 8.9 Å. (C) Valine is the preferred residue at position 61. A sequence logo was constructed of Block B from the 50 representative Prp8 inteins (S1 Fig). This shows absolute conservation of the histidine (position 10) and a strong preference for threonine (position 7) in the TxxH motif. However, the Block B cysteine (position 6, red box) is not highly conserved across Prp8 inteins, and most encode valine at this site. *Cne*, *C*. *neoformans*; GFP, green fluorescent protein; LE, ligated exteins; MIG, MBP-Intein-GFP; P, precursor; Prp8, pre-mRNA processing factor 8
(TIF)

S8 Fig Copper inhibition of MIG Prp8 A-1V C61 mutants. (A) Mutations to C61 in MIG Prp8 A-1V slow down splicing. The B block C61 was mutated to valine (C61V), alanine (C61A), and serine (C61S), and splicing was observed over time in MIG. Initial splicing rates were determined by calculating the loss of precursor over time (P_t0_−P_t1_/60 min) with standard error and are as follows: WT, (1.01 ± 0.07) × 10^−1^% per min; C61V, (1.07 ± 0.08) × 10^−1^% per min; C61A, (6.22 ± 0.50) × 10^−2^% per min, and C61S, (2.92 ± 1.04) × 10^−2^% per min. The C61V mutant splices similarly to WT, whereas C61A and C61S are slower. A quantitation is shown to the right with the amount of precursor (P) at each time point. Data are representative of 3 biological replicates and mean standard deviations are shown. Trend lines are fit to show the decay curve. Data available in S1 Data. (B) MIG Prp8 A-1V B block cysteine mutants are inhibited by copper. To test whether copper inhibition was caused by C1 oxidation, C61 mutants were treated with CuSO_4_. After induction of MIG, the cells were lysed, and 1 mM CuSO_4_ was added. The lysates were incubated at 30°C, and aliquots were collected at the indicated time. Samples were run on SDS-PAGE and scanned for GFP fluorescence. None of the C61 mutants show an increase in LE over time, with little loss of precursor (P). This indicates that at least C1 oxidation by copper is sufficient to cause the observed splicing inhibition and that disulfide bonds are not involved. Quantitation is shown below in a stacked plot. Data are representative of 3 biological replicates, and mean standard deviations are shown. Data available in S1 Data. GFP, green fluorescent protein; LE, ligated exteins; MIG, MBP-Intein-GFP; Prp8, pre-mRNA processing factor 8; WT, wild type.
(TIF)

S9 Fig Mass spectrometry of cysteine modifications. (A) Intact *Cne* Prp8 intein shows small mass shift. Purified *Cne* Prp8 intein was untreated or treated with 10× excess copper and separated and analyzed using LC-MS. The peaks were deconvoluted, and the expected mass of the Prp8 intein, 19,588 Da, is seen as the largest peak. A small, 32 Da shift (19,620 Da) was visible with both no treatment and copper treatment only (arrow). This suggests that highly reactive cysteines are modified by atmospheric oxygen alone. (B) C1 and C61 are oxidized with copper treatment. Trypsin-digested fragments of copper-treated *Cne* Prp8 intein were separated and sprayed using LC-MS/MS (insets). Peptides (red peaks) containing C1 or C61 were detected and further analyzed using multiple reaction MIDAS to confirm the identity and location of oxidation. The chromatogram shows elution time for both cysteines consistent with a single additional oxygen or a sulfenic acid modification. *Cne*, *C*. *neoformans*; LC-MS, liquid chromatography-mass spectrometry; LC-MS/MS, liquid chromatography-mass spectrometry/mass spectrometry; MIDAS, monitoring-initiated detection and sequencing; Prp8, pre-mRNA processing factor 8
(TIF)

S10 Fig Mapping of Prp8 intein insertion sites to Prp8 extein domains. The 7 unique insertion sites (a–g) were mapped to a solved structure of Prp8 from a *S*. *cerevisiae* C complex spliceosome (PDB 5GMK, chain A from Wan and colleagues, 2016) by locating the +1 residue. This Prp8 structure was used because the insertion sites are all resolved. The +1 residues are shown as red spheres and labeled a through g. Most Prp8 inteins localize close to the active center of Prp8. Some insertions are in the N-terminal domain, which provides structural integrity to the spliceosome. A corresponding line diagram of Prp8 exteins shows the domains of the host protein from amino acid residues 127 to 2084 with arrows indicating the site of intein insertion with the residue number and insertion site letter. The domains are as follows: N-terminal domain, gray; RT Palm/Finger, dark blue; Thumb/X, light blue; linker, green; endonuclease, yellow; and RNase H-like, orange. PDB, Protein Data Bank; Prp8, pre-mRNA processing factor 8
(TIF)

S11 Fig Model of the *Cne* Prp8 intein interrupting Prp8 and the spliceosomal U4/U6.U5 tri-snRNP. The Prp8 intein-containing Prp8 precursor model was docked into a cryo-EM tri-snRNP structure from *Sce* (PDB 5GAN) to look for intein-spliceosome disruptions. Prp8 is shown as lavender, and the Prp8 intein is shown as red, and the rest of the tri-snRP components are colored by chain. This reveals that the Prp8 intein would occupy a relatively crowded, centralized location of the tri-snRNP (circled). The intein clashes are shown here (with labels) and noted in Fig 7B. *Cne*, *C*. *neoformans*; cryo-EM, cryogenic electron microscopy; PDB, Protein Data Bank; Prp8, pre-mRNA processing factor 8; *Sce*, *S*. *cerevisiae*; tri-snRNP, triple small nuclear ribonucleoprotein.
(TIF)

S1 Table Bacterial and fungal strains. A list of bacterial strains used for various cloning, overexpression, and purification studies is provided. Strains of fungi and yeast used for in vivo studies are also listed.
(DOCX)

S2 Table Plasmids and constructs. A list of MIG constructs and purification vectors with corresponding backbones are provided. MIG, MBP-Intein-GFP.
(DOCX)

S3 Table Oligonucleotide primers. Primers used for the construction of various vectors or for the mutation of plasmids are provided.
(DOCX)

S4 Table Crystallization information. Data collection, refinement statistics, and model details for (A) the unbound and (B) the Zn^2+^-bound *Cne* Prp8 intein crystal structures. *Cne*, *C*. *neoformans*; Prp8, pre-mRNA processing factor 8
(DOCX)

S1 Data MIG Prp8 quantitation. Individual numerical values that underlie any graphs (Figs 4B, 4C, 5A, 5B, and S6B, S7A, S8A and S8B Figs) are provided in separate sheets. Values were calculated from biological triplicate gel images using ImageJ software. Levels of precursor (P), LE, and OPC products are given out of a total of 100. Some graphs use percent precursor as a proxy for splicing. Time points are indicated. LE, ligated exteins; MIG, MBP-Intein-GFP; OPC, off-pathway cleavage; Prp8, pre-mRNA processing factor 8
(XLSX)

## Abbreviations

ABNR: Adapted-Basis Newton-Raphson
Cne: Cryptococcus neoformans
CT: carboxy terminus
Dme: Drosophila melanogaster
EDTA: ethylenediaminetetraacetic acid
EPI: enhanced production
GFP: green fluorescent protein
HHc: Hedgehog C-terminal domain
IDA: information dependent acquisition
ITC: isothermal titration calorimetry
LB: Luria Broth
LC: liquid chromatography
LE: ligated exteins
NT: amino terminus
MIDAS: monitoring-initiated detection and sequencing
MIG: MBP-Intein-GFP
MBP: maltose binding protein
MRM: multiple reaction monitoring
mya: millon years ago
NJ: neighbor-joining
OPC: off-pathway cleavage
PBST: PBS with Tween-20
PDB: Protein Data Bank
Prp8: pre-mRNA processing factor 8
RMSD: Root-mean-square deviation
RNS: reactive nitrogen species
ROS: reactive oxygen species
SC: synthetic complete media
Sce: Saccharomyces cerevisiae
SD: steepest descent
TCEP: tris-(2-carboxyethyl)phosphine
tri-snRNP: triple small nuclear ribonucleoprotein

## References

[1] Grainger RJ, Beggs JD. Prp8 protein: at the heart of the spliceosome. Rna. 2005;11(5):533–57. 10.1261/rna.2220705 15840809 PMC1370742

[2] Mayerle M, Guthrie C. Prp8 retinitis pigmentosa mutants cause defects in the transition between the catalytic steps of splicing. Rna. 2016;22(5):793–809. 10.1261/rna.055459.115 26968627 PMC4836653

[3] Daiger SP, Sullivan LS, Bowne SJ. Genes and mutations causing retinitis pigmentosa. Clinical genetics. 2013;84(2):132–41. 10.1111/cge.12203 23701314 PMC3856531

[4] Qu G, Kaushal PS, Wang J, Shigematsu H, Piazza CL, Agrawal RK, et al Structure of a group II intron in complex with its reverse transcriptase. Nature structural & molecular biology. 2016;23(6):549–57. 10.1038/nsmb.3220 27136327 PMC4899178

[5] Galej WP, Oubridge C, Newman AJ, Nagai K. Crystal structure of Prp8 reveals active site cavity of the spliceosome. Nature. 2013;493(7434):638–43. 10.1038/nature11843 23354046 PMC3672837

[6] Dlakic M, Mushegian A. Prp8, the pivotal protein of the spliceosomal catalytic center, evolved from a retroelement-encoded reverse transcriptase. Rna. 2011;17(5):799–808. 10.1261/rna.2396011 21441348 PMC3078730

[7] Zhan X, Yan C, Zhang X, Lei J, Shi Y. Structure of a human catalytic step I spliceosome. Science. 2018;359(6375):537–45. 10.1126/science.aar6401 . 29301961

[8] Wilkinson ME, Fica SM, Galej WP, Norman CM, Newman AJ, Nagai K. Postcatalytic spliceosome structure reveals mechanism of 3'-splice site selection. Science. 2017;358(6368):1283–8. 10.1126/science.aar3729 29146871 PMC5808836

[9] Bai R, Wan R, Yan C, Lei J, Shi Y. Structures of the fully assembled Saccharomyces cerevisiae spliceosome before activation. Science. 2018;360(6396):1423–9. 10.1126/science.aau0325 . 29794219

[10] Wan R, Yan C, Bai R, Huang G, Shi Y. Structure of a yeast catalytic step I spliceosome at 3.4 A resolution. Science. 2016;353(6302):895–904. 10.1126/science.aag2235 . 27445308

[11] Wan R, Yan C, Bai R, Wang L, Huang M, Wong CC, et al The 3.8 A structure of the U4/U6.U5 tri-snRNP: Insights into spliceosome assembly and catalysis. Science. 2016;351(6272):466–75. 10.1126/science.aad6466 . 26743623

[12] Yan C, Hang J, Wan R, Huang M, Wong CC, Shi Y. Structure of a yeast spliceosome at 3.6-angstrom resolution. Science. 2015;349(6253):1182–91. 10.1126/science.aac7629 . 26292707

[13] Saleh L, Perler FB. Protein splicing in cis and in trans. Chemical record. 2006;6(4):183–93. 10.1002/tcr.20082 . 16900466

[14] Novikova O, Topilina N, Belfort M. Enigmatic distribution, evolution, and function of inteins. The Journal of biological chemistry. 2014;289(21):14490–7. 10.1074/jbc.R114.548255 24695741 PMC4031506

[15] Perler FB, Olsen GJ, Adam E. Compilation and analysis of intein sequences. Nucleic acids research. 1997;25(6):1087–93. 10.1093/nar/25.6.1087 9092614 PMC146560

[16] Gimble FS, Thorner J. Homing of a DNA endonuclease gene by meiotic gene conversion in Saccharomyces cerevisiae. Nature. 1992;357(6376):301–6. 10.1038/357301a0 . 1534148

[17] Liu XQ. Protein-splicing intein: Genetic mobility, origin, and evolution. Annual review of genetics. 2000;34:61–76. 10.1146/annurev.genet.34.1.61 . 11092822

[18] Lennon CW, Stanger M, Banavali NK, Belfort M. Conditional Protein Splicing Switch in Hyperthermophiles through an Intein-Extein Partnership. mBio. 2018;9(1). 10.1128/mBio.02304-17 29382734 PMC5790916

[19] Lennon CW, Stanger M, Belfort M. Protein splicing of a recombinase intein induced by ssDNA and DNA damage. Genes & development. 2016;30(24):2663–8. Epub 2016/12/30. 10.1101/gad.289280.116 28031248 PMC5238726

[20] Kelley DS, Lennon CW, Sea P, Belfort M, Novikova O. Mycobacteriophages as Incubators for Intein Dissemination and Evolution. mBio. 2016;7(5). 10.1128/mBio.01537-16 27703073 PMC5050341

[21] Topilina NI, Green CM, Jayachandran P, Kelley DS, Stanger MJ, Piazza CL, et al SufB intein of Mycobacterium tuberculosis as a sensor for oxidative and nitrosative stresses. Proceedings of the National Academy of Sciences of the United States of America. 2015;112(33):10348–53. Epub 2015/08/05. 10.1073/pnas.1512777112 . 26240361 PMC4547236

[22] Nicastri MC, Xega K, Li L, Xie J, Wang C, Linhardt RJ, et al Internal disulfide bond acts as a switch for intein activity. Biochemistry. 2013;52(34):5920–7. Epub 2013/08/03. 10.1021/bi400736c 23906287 PMC3801215

[23] Reitter JN, Cousin CE, Nicastri MC, Jaramillo MV, Mills KV. Salt-dependent conditional protein splicing of an intein from halobacterium salinarum. Biochemistry. 2016;55(9):1279–82. Epub 2016/02/26. 10.1021/acs.biochem.6b00128 . 26913597

[24] Topilina NI, Novikova O, Stanger M, Banavali NK, Belfort M. Post-translational environmental switch of RadA activity by extein-intein interactions in protein splicing. Nucleic acids research. 2015;43(13):6631–48. Epub 2015/06/24. 10.1093/nar/gkv612 . 26101259 PMC4513877

[25] Kelley DS, Lennon C. W., Li Z., Miller M. R., Banavali N. K., Li H., et al Mycobacterial DnaB helicase intein as oxidative stress sensor. Nature Communications. 2018; 9(4363). 10.1038/s41467-018-06554-x PMC6195587 30341292

[26] Novikova O, Jayachandran P, Kelley DS, Morton Z, Merwin S, Topilina NI, et al Intein clustering suggests functional importance in different domains of life. Molecular biology and evolution. 2016;33(3):783–99. Epub 2015/11/27. 10.1093/molbev/msv271 26609079 PMC4760082

[27] Green CM, Novikova O, Belfort M. The dynamic intein landscape of eukaryotes. Mobile DNA. 2018;9:4 10.1186/s13100-018-0111-x 29416568 PMC5784728

[28] Theodoro RC, Bagagli E. Inteins in pathogenic fungi: a phylogenetic tool and perspectives for therapeutic applications. Memorias do Instituto Oswaldo Cruz. 2009;104(3):497–504. 10.1590/s0074-02762009000300017 . 19547879

[29] Janbon G. Introns in Cryptococcus. Memorias do Instituto Oswaldo Cruz. 2018;113(7):e170519 10.1590/0074-02760170519 29513783 PMC5851062

[30] Galej WP, Nguyen TH, Newman AJ, Nagai K. Structural studies of the spliceosome: zooming into the heart of the machine. Current opinion in structural biology. 2014;25:57–66. 10.1016/j.sbi.2013.12.002 24480332 PMC4045393

[31] Elleuche SaP S. Fungal inteins: distribution, evolution, and applications. In: Anke T, Schuffler A., editor. The Mycota. 152018. p. 57–85.

[32] Stajich JE, Berbee ML, Blackwell M, Hibbett DS, James TY, Spatafora JW, et al The fungi. Current biology: CB. 2009;19(18):R840–5. 10.1016/j.cub.2009.07.004 19788875 PMC2913116

[33] Poulter RT, Goodwin TJ, Butler MI. The nuclear-encoded inteins of fungi. Fungal genetics and biology: FG & B. 2007;44(3):153–79. 10.1016/j.fgb.2006.07.012 . 17046294

[34] Pietrokovski S. Modular organization of inteins and C-terminal autocatalytic domains. Protein science: a publication of the Protein Society. 1998;7(1):64–71. 10.1002/pro.5560070106 9514260 PMC2143824

[35] Butler MI, Gray J, Goodwin TJ, Poulter RT. The distribution and evolutionary history of the PRP8 intein. BMC evolutionary biology. 2006;6:42 10.1186/1471-2148-6-42 16737526 PMC1508164

[36] Monier A, Sudek S, Fast NM, Worden AZ. Gene invasion in distant eukaryotic lineages: discovery of mutually exclusive genetic elements reveals marine biodiversity. The ISME journal. 2013;7(9):1764–74. 10.1038/ismej.2013.70 23635865 PMC3749507

[37] Urushihara H, Kuwayama H, Fukuhara K, Itoh T, Kagoshima H, Shin IT, et al Comparative genome and transcriptome analyses of the social amoeba Acytostelium subglobosum that accomplishes multicellular development without germ-soma differentiation. BMC genomics. 2015;16:80 10.1186/s12864-015-1278-x 25758444 PMC4334915

[38] Volkmann G, Mootz HD. Recent progress in intein research: from mechanism to directed evolution and applications. Cellular and molecular life sciences: CMLS. 2013;70(7):1185–206. 10.1007/s00018-012-1120-4 . 22926412 PMC11113529

[39] Werner E, Wende W, Pingoud A, Heinemann U. High resolution crystal structure of domain I of the Saccharomyces cerevisiae homing endonuclease PI-SceI. Nucleic acids research. 2002;30(18):3962–71. 10.1093/nar/gkf523 12235380 PMC137108

[40] Moure CM, Gimble FS, Quiocho FA. Crystal structure of the intein homing endonuclease PI-SceI bound to its recognition sequence. Nature structural biology. 2002;9(10):764–70. 10.1038/nsb840 . 12219083

[41] Dearden AK, Callahan B, Roey PV, Li Z, Kumar U, Belfort M, et al A conserved threonine spring-loads precursor for intein splicing. Protein science: a publication of the Protein Society. 2013;22(5):557–63. 10.1002/pro.2236 23423655 PMC3649257

[42] Mills KV, Johnson MA, Perler FB. Protein splicing: how inteins escape from precursor proteins. The Journal of biological chemistry. 2014;289(21):14498–505. Epub 2014/04/04. 10.1074/jbc.R113.540310 24695729 PMC4031507

[43] Hall TM, Porter JA, Young KE, Koonin EV, Beachy PA, Leahy DJ. Crystal structure of a Hedgehog autoprocessing domain: homology between Hedgehog and self-splicing proteins. Cell. 1997;91(1):85–97. 10.1016/s0092-8674(01)80011-8 . 9335337

[44] Burglin TR. The Hedgehog protein family. Genome biology. 2008;9(11):241 10.1186/gb-2008-9-11-241 19040769 PMC2614485

[45] Perler FB. Protein splicing of inteins and hedgehog autoproteolysis: structure, function, and evolution. Cell. 1998;92(1):1–4. 10.1016/s0092-8674(00)80892-2 . 9489693

[46] Roelink H. The Hedgehog signaling domain was acquired from a prokaryote. bioRxiv2018.

[47] Van Roey P, Pereira B, Li Z, Hiraga K, Belfort M, Derbyshire V. Crystallographic and mutational studies of Mycobacterium tuberculosis recA mini-inteins suggest a pivotal role for a highly conserved aspartate residue. Journal of molecular biology. 2007;367(1):162–73. 10.1016/j.jmb.2006.12.050 17254599 PMC1852430

[48] Pearl EJ, Bokor AA, Butler MI, Poulter RT, Wilbanks SM. Preceding hydrophobic and beta-branched amino acids attenuate splicing by the CnePRP8 intein. Biochimica et biophysica acta. 2007;1774(8):995–1001. 10.1016/j.bbapap.2007.05.015 . 17604706

[49] Amitai G, Callahan BP, Stanger MJ, Belfort G, Belfort M. Modulation of intein activity by its neighboring extein substrates. Proceedings of the National Academy of Sciences of the United States of America. 2009;106(27):11005–10. 10.1073/pnas.0904366106 19541659 PMC2708771

[50] Weiss G, Schaible UE. Macrophage defense mechanisms against intracellular bacteria. Immunological reviews. 2015;264(1):182–203. 10.1111/imr.12266 25703560 PMC4368383

[51] Missall TA, Lodge JK, McEwen JE. Mechanisms of resistance to oxidative and nitrosative stress: implications for fungal survival in mammalian hosts. Eukaryotic cell. 2004;3(4):835–46. 10.1128/EC.3.4.835-846.2004 15302816 PMC500878

[52] Chan H, Pearson CS, Green CM, Li Z, Zhang J, Belfort G, et al Exploring Intein Inhibition by Platinum Compounds as an Antimicrobial Strategy. The Journal of biological chemistry. 2016;291(43):22661–70. Epub 2016/09/10. 10.1074/jbc.M116.747824 27609519 PMC5077202

[53] Zhang L, Xiao N, Pan Y, Zheng Y, Pan Z, Luo Z, et al Binding and inhibition of copper ions to RecA inteins from Mycobacterium tuberculosis. Chemistry. 2010;16(14):4297–306. 10.1002/chem.200903584 . 20209535

[54] Zhang L, Zheng Y, Xi Z, Luo Z, Xu X, Wang C, et al Metal ions binding to recA inteins from Mycobacterium tuberculosis. Molecular bioSystems. 2009;5(6):644–50. 10.1039/b903144h 19462022 PMC2790073

[55] Sun P, Ye S, Ferrandon S, Evans TC, Xu MQ, Rao Z. Crystal structures of an intein from the split dnaE gene of Synechocystis sp. PCC6803 reveal the catalytic model without the penultimate histidine and the mechanism of zinc ion inhibition of protein splicing. Journal of molecular biology. 2005;353(5):1093–105. 10.1016/j.jmb.2005.09.039 . 16219320

[56] Mills KV, Paulus H. Reversible inhibition of protein splicing by zinc ion. The Journal of biological chemistry. 2001;276(14):10832–8. 10.1074/jbc.M011149200 . 11152694

[57] Nichols NM, Benner JS, Martin DD, Evans TC Jr., Zinc ion effects on individual Ssp DnaE intein splicing steps: regulating pathway progression. Biochemistry. 2003;42(18):5301–11. 10.1021/bi020679e . 12731871

[58] Giles NM, Watts AB, Giles GI, Fry FH, Littlechild JA, Jacob C. Metal and redox modulation of cysteine protein function. Chemistry & biology. 2003;10(8):677–93. . 12954327 10.1016/s1074-5521(03)00174-1

[59] Kelley DS, Lennon CW, Li Z, Miller MR, Banavali NK, Li H, et al Mycobacterial DnaB helicase intein as oxidative stress sensor. Nat Commun. 2018;9(1):4363 10.1038/s41467-018-06554-x . 30341292 PMC6195587

[60] Callahan BP, Topilina NI, Stanger MJ, Van Roey P, Belfort M. Structure of catalytically competent intein caught in a redox trap with functional and evolutionary implications. Nature structural & molecular biology. 2011;18(5):630–3. 10.1038/nsmb.2041 21460844 PMC3087850

[61] Nguyen THD, Galej WP, Bai XC, Oubridge C, Newman AJ, Scheres SHW, et al Cryo-EM structure of the yeast U4/U6.U5 tri-snRNP at 3.7 A resolution. Nature. 2016;530(7590):298–302. 10.1038/nature16940 26829225 PMC4762201

[62] Shi Y. The Spliceosome: A Protein-Directed Metalloribozyme. Journal of molecular biology. 2017;429(17):2640–53. 10.1016/j.jmb.2017.07.010 . 28733144

[63] Schreib CC, Bowman EK, Hernandez CA, Lucas AL, Potts CHS, Maeder C. Functional and Biochemical Characterization of Dib1's Role in Pre-Messenger RNA Splicing. Journal of molecular biology. 2018;430(11):1640–51. 10.1016/j.jmb.2018.04.027 29715471 PMC6155989

[64] Walker JE, Saraste M, Runswick MJ, Gay NJ. Distantly related sequences in the alpha- and beta-subunits of ATP synthase, myosin, kinases and other ATP-requiring enzymes and a common nucleotide binding fold. The EMBO journal. 1982;1(8):945–51. 6329717 10.1002/j.1460-2075.1982.tb01276.x PMC553140

[65] Aranko AS, Wlodawer A, Iwai H. Nature's recipe for splitting inteins. Protein engineering, design & selection: PEDS. 2014;27(8):263–71. 10.1093/protein/gzu028 25096198 PMC4133565

[66] Zhang L, Zheng Y, Callahan B, Belfort M, Liu Y. Cisplatin inhibits protein splicing, suggesting inteins as therapeutic targets in mycobacteria. The Journal of biological chemistry. 2011;286(2):1277–82. Epub 2010/11/10. M110.171124 [pii] 10.1074/jbc.M110.171124 21059649 PMC3020735

[67] Paulus H. Inteins as targets for potential antimycobacterial drugs. Frontiers in bioscience: a journal and virtual library. 2003;8:s1157–65. 10.2741/1195 . 12957838

[68] Li Z, Fu B, Green CM, Liu B, Zhang J, Lang Y, et al Cisplatin protects mice from challenge of Cryptococcus neoformans by targeting the Prp8 intein. Emerging Microbes & Infections. 2019, in press. 10.1080/22221751.2019.1625727 PMC6598491 31223062

[69] Fairlamb AH, Gow NA, Matthews KR, Waters AP. Drug resistance in eukaryotic microorganisms. Nature microbiology. 2016;1(7):16092 10.1038/nmicrobiol.2016.92 27572976 PMC5215055

[70] Chong S, Williams KS, Wotkowicz C, Xu MQ. Modulation of protein splicing of the Saccharomyces cerevisiae vacuolar membrane ATPase intein. The Journal of biological chemistry. 1998;273(17):10567–77. 10.1074/jbc.273.17.10567 . 9553117

[71] Lew BM, Paulus H. An in vivo screening system against protein splicing useful for the isolation of non-splicing mutants or inhibitors of the RecA intein of Mycobacterium tuberculosis. Gene. 2002;282(1–2):169–77. 10.1016/s0378-1119(01)00836-8 . 11814689

[72] Ballou ER, Wilson D. The roles of zinc and copper sensing in fungal pathogenesis. Current opinion in microbiology. 2016;32:128–34. 10.1016/j.mib.2016.05.013 27327380 PMC4992176

[73] Smith AD, Logeman BL, Thiele DJ. Copper Acquisition and Utilization in Fungi. Annual review of microbiology. 2017;71:597–623. 10.1146/annurev-micro-030117-020444 . 28886682 PMC6827982

[74] Hodgkinson V, Petris MJ. Copper homeostasis at the host-pathogen interface. The Journal of biological chemistry. 2012;287(17):13549–55. 10.1074/jbc.R111.316406 22389498 PMC3340201

[75] Dos Santos FM, Piffer AC, Schneider RO, Ribeiro NS, Garcia AWA, Schrank A, et al Alterations of zinc homeostasis in response to Cryptococcus neoformans in a murine macrophage cell line. Future microbiology. 2017;12:491–504. 10.2217/fmb-2016-0160 . 28492340

[76] Bergkessel M, Whitworth GB, Guthrie C. Diverse environmental stresses elicit distinct responses at the level of pre-mRNA processing in yeast. Rna. 2011;17(8):1461–78. 10.1261/rna.2754011 21697354 PMC3153971

[77] Gonzalez-Hilarion S, Paulet D, Lee KT, Hon CC, Lechat P, Mogensen E, et al Intron retention-dependent gene regulation in Cryptococcus neoformans. Scientific reports. 2016;6:32252 10.1038/srep32252 27577684 PMC5006051

[78] Sievers F, Wilm A, Dineen D, Gibson TJ, Karplus K, Li W, et al Fast, scalable generation of high-quality protein multiple sequence alignments using Clustal Omega. Molecular systems biology. 2011;7:539 10.1038/msb.2011.75 21988835 PMC3261699

[79] Kumar S, Stecher G, Tamura K. MEGA7: Molecular Evolutionary Genetics Analysis Version 7.0 for Bigger Datasets. Molecular biology and evolution. 2016;33(7):1870–4. 10.1093/molbev/msw054 . 27004904 PMC8210823

[80] Sitnikova T, Rzhetsky A, Nei M. Interior-branch and bootstrap tests of phylogenetic trees. Molecular biology and evolution. 1995;12(2):319–33. 10.1093/oxfordjournals.molbev.a040205 . 7700156

[81] Crooks GE, Hon G, Chandonia JM, Brenner SE. WebLogo: a sequence logo generator. Genome research. 2004;14(6):1188–90. 10.1101/gr.849004 15173120 PMC419797

[82] Li Z, Brecher M, Deng YQ, Zhang J, Sakamuru S, Liu B, et al Existing drugs as broad-spectrum and potent inhibitors for Zika virus by targeting NS2B-NS3 interaction. Cell research. 2017;27(8):1046–64. 10.1038/cr.2017.88 28685770 PMC5539352

[83] Unwin RD, Griffiths JR, Whetton AD. A sensitive mass spectrometric method for hypothesis-driven detection of peptide post-translational modifications: multiple reaction monitoring-initiated detection and sequencing (MIDAS). Nature protocols. 2009;4(6):870–7. 10.1038/nprot.2009.57 . 19444244

[84] Otwinowski Z, Minor W. [20] Processing of X-ray diffraction data collected in oscillation mode. Methods in enzymology. 1997;276:307–26. 10.1016/S0076-6879(97)76066-X. . 27754618

[85] Adams PD, Afonine PV, Bunkoczi G, Chen VB, Davis IW, Echols N, et al PHENIX: a comprehensive Python-based system for macromolecular structure solution. Acta crystallographica Section D, Biological crystallography. 2010;66(Pt 2):213–21. 10.1107/S0907444909052925 20124702 PMC2815670

[86] Emsley P, Lohkamp B, Scott WG, Cowtan K. Features and development of Coot. Acta crystallographica Section D, Biological crystallography. 2010;66(Pt 4):486–501. Epub 2010/04/13. 10.1107/S0907444910007493 20383002 PMC2852313

[87] Arras SD, Chitty JL, Blake KL, Schulz BL, Fraser JA. A genomic safe haven for mutant complementation in Cryptococcus neoformans. PLoS ONE. 2015;10(4):e0122916 10.1371/journal.pone.0122916 25856300 PMC4391909

[88] Needleman SB, Wunsch C. D. A general method applicable to the search for similarities in the amino acid sequence of two proteins. Journal of molecular biology. 1970;48:443–53. 10.1016/0022-2836(70)90057-4 5420325

[89] Rice P, Longden I, Bleasby A. EMBOSS: the European Molecular Biology Open Software Suite. Trends in genetics: TIG. 2000;16(6):276–7. 10.1016/s0168-9525(00)02024-2 . 10827456

[90] Sali A, Blundell TL. Comparative protein modelling by satisfaction of spatial restraints. Journal of molecular biology. 1993;234(3):779–815. 10.1006/jmbi.1993.1626 . 8254673

[91] Shen MY, Sali A. Statistical potential for assessment and prediction of protein structures. Protein science: a publication of the Protein Society. 2006;15(11):2507–24. 10.1110/ps.062416606 17075131 PMC2242414

[92] Melo F, Sanchez R, Sali A. Statistical potentials for fold assessment. Protein science: a publication of the Protein Society. 2002;11(2):430–48. 10.1002/pro.110430 11790853 PMC2373452

[93] Humphrey W, Dalke A, Schulten K. VMD: visual molecular dynamics. Journal of molecular graphics. 1996;14(1):33–8, 27–8. 8744570 10.1016/0263-7855(96)00018-5

[94] Brooks B, Bruccoleri R., Olafson B., Swaminathan S., Karplus M. CHARMM: A program for macromolecular energy, minimization, and dynamics calculations. Journal of computational chemistry. 1983;(4):187–217.

[95] Brooks BR, Brooks CL 3rd, Mackerell AD, Jr., Nilsson L, Petrella RJ, Roux B, et al CHARMM: the biomolecular simulation program. Journal of computational chemistry. 2009;30(10):1545–614. 10.1002/jcc.21287 19444816 PMC2810661

[96] Best RB, Zhu X, Shim J, Lopes PE, Mittal J, Feig M, et al Optimization of the additive CHARMM all-atom protein force field targeting improved sampling of the backbone phi, psi and side-chain chi(1) and chi(2) dihedral angles. Journal of chemical theory and computation. 2012;8(9):3257–73. 10.1021/ct300400x 23341755 PMC3549273

[97] Ryckaert J-P, Ciccotti G., Berendsen H. J. Numerical integration of the cartesian equations of motion of a system with constraints: molecular dyanimcs of n-alkanes. Journal of Computational Physics. 1997;23:327–41.

