# Supplementary figures and images for: Spliceosomal Prp8 intein at the crossroads of protein and RNA splicing
Source: PLoS Biol. 2019 Oct 10;17(10):e3000104. doi: 10.1371/journal.pbio.3000104 (PMC6805012; doi:10.1371/journal.pbio.3000104)

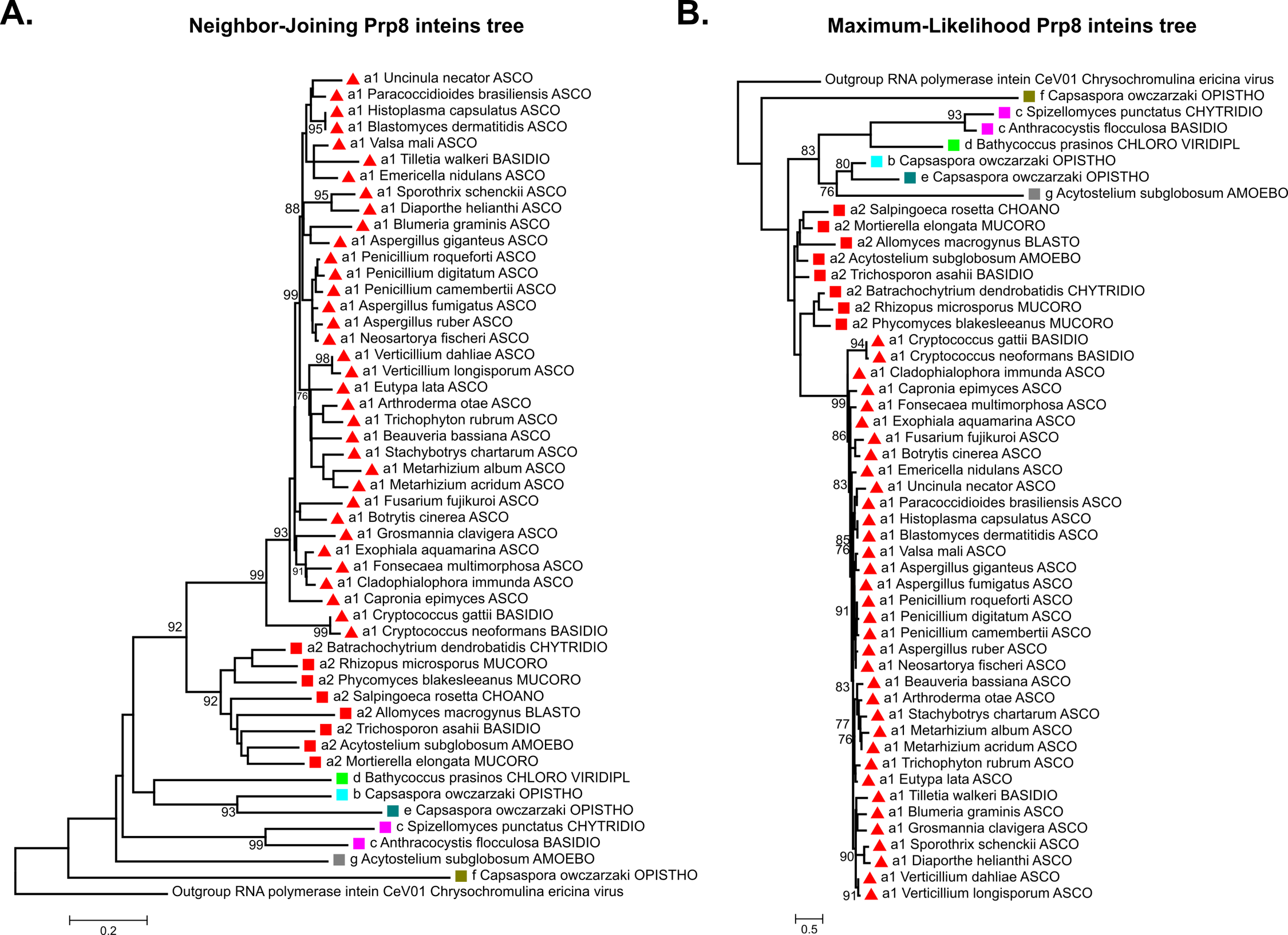

Supplement: S1 Fig — (A) A phylogenetic tree of Prp8 inteins was reconstructed based on an amino acid multiple sequence alignment of the splicing blocks (A, B, F, G) using the NJ algorithm and an interior-branch test with 1,000 replicates. Fifty representatives covering Prp8 intein diversity were selected, and the full name of each intein-containing organism is listed. Colored symbols represent the insertion site and correspond to colors in Fig 1A. Letters (a1, a2, b, c, d, e, f, g) represent each of the 7 unique insertion sites. (B) A phylogenetic tree of Prp8 inteins was reconstructed based on an amino acid multiple sequence alignment of the splicing blocks (A, B, F, G) using the ML method and evaluated with SH-aLRT. The substitution model, WAG+G+I, was selected using ProtTest 3 (https://github.com/ddarriba/prottest3). ML tree follows the same formatting as in panel A and shows similar architecture as NJ tree. Amoebo, Amoebozoa; Asco, Ascomycota; Basidio, Basidiomycota; Blasto, Blastocladiomycota; Choano, Choanoflagellida; Chloro Viridipl, Chlorophyta Viridiplantae; Chytridio, Chytridiomycota; ML, maximum likelihood; Mucoro, Mucoromycota; NJ, neighbor-joining; Opistho, Opisthokonta; Prp8, pre-mRNA processing factor 8; SH-aLRT, Shimodaira–Hasegawa nonparametric approximate likelihood-ratio test (TIF) [file pbio.3000104.s001.tif]

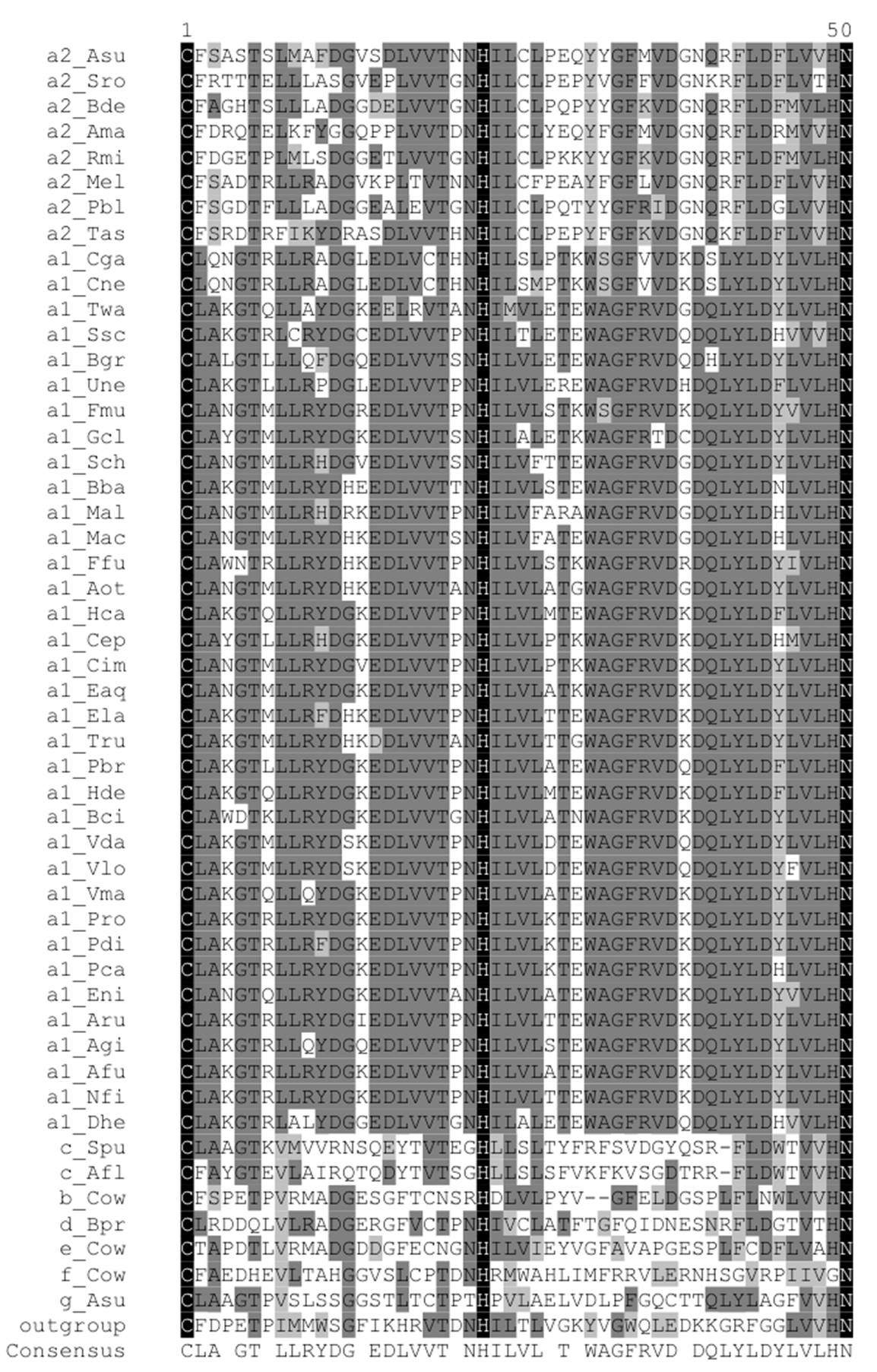

Supplement: S2 Fig — Comparative analysis of amino acid residues found in Blocks A, B, F, and G from the selected 50 representative Prp8 inteins, shown with abbreviated species names (full names in S1 Fig). Letters (a1, a2, b, c, d, e, f, g) represent each of the 7 unique insertion sites. Shading is as follows: black, identical amino acid; dark gray, conserved amino acid; light gray, similar amino acid substitution. Prp8, pre-mRNA processing factor 8 (TIF) [file pbio.3000104.s002.tif]

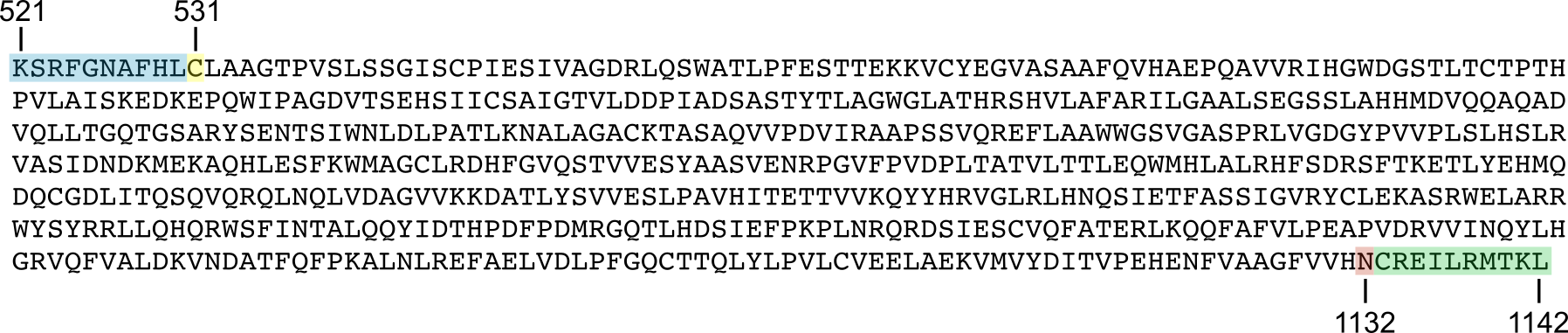

Supplement: S3 Fig — In the amoeba Asu, an intein was identified at a new site in Prp8, here termed g. This is the seventh site in which a Prp8 intein has been found. The full site g intein sequence is shown, plus 10 flanking N-extein (blue) and C-extein (green) amino acids. The Asu C1 (yellow) and terminal asparagine (red) are highlighted. Residue numbering corresponds to the Asu Prp8 exteins. Accession number: XP_0127532. Asu, Acytostelium subglobosum; Prp8, pre-mRNA processing factor 8 (TIF) [file pbio.3000104.s003.tif]

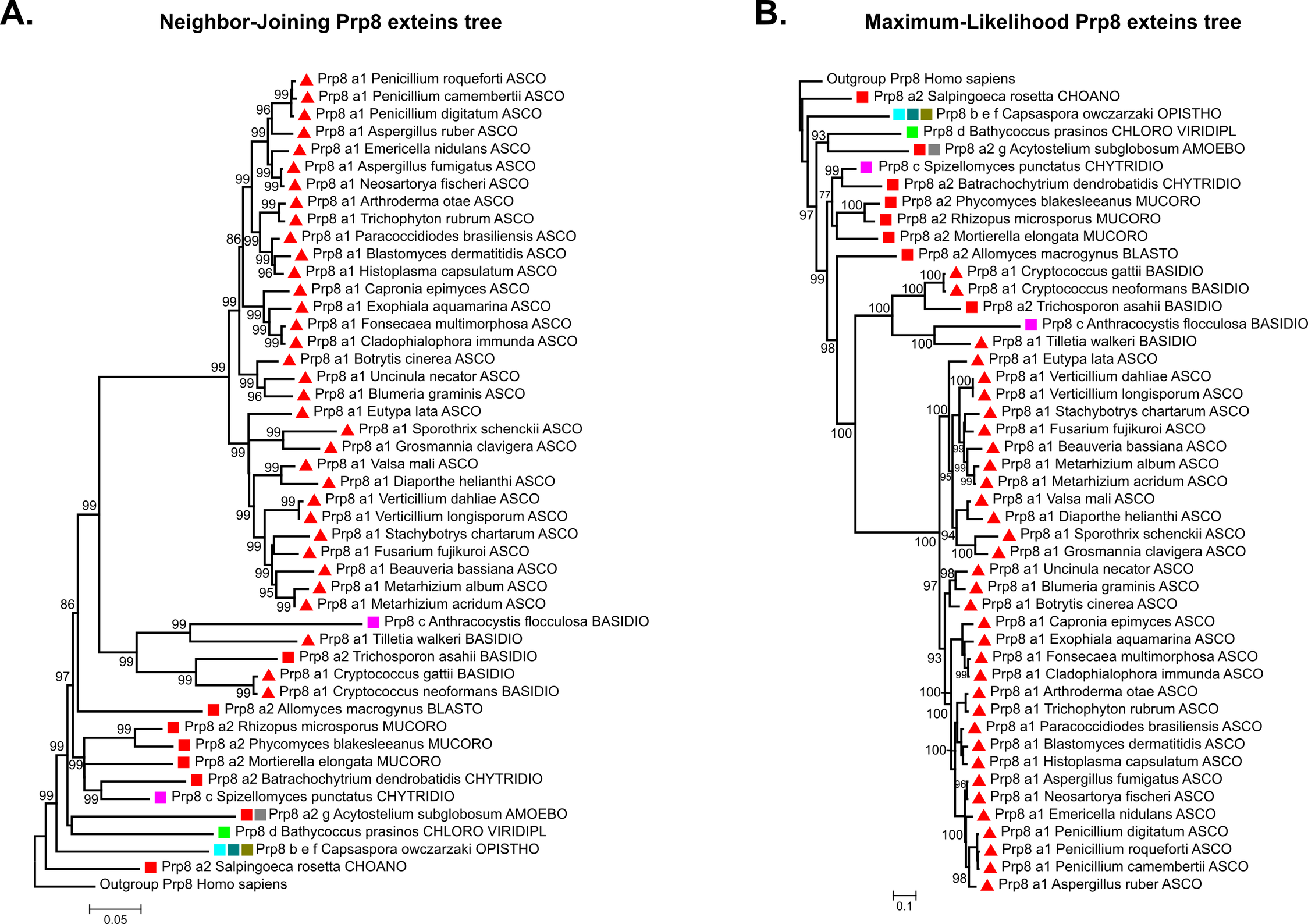

Supplement: S4 Fig — (A) A phylogenetic tree of Prp8 exteins corresponding to inteins (see S1 Fig) was reconstructed based on an amino acid multiple sequence alignment using the NJ algorithm and an interior-branch test with 1,000 replicates. Extreme conservation among Prp8 exteins is observed along with grouping by host organism phylogeny. Colored symbols represent the intein insertion site of the exteins and correspond to colors in Fig 1A. Letters (a1, a2, b, c, d, e, f, and g) represent each of the 7 unique insertion sites. Phylum abbreviations are listed in the S1 Fig legend. (B) A phylogenetic tree of Prp8 exteins was reconstructed based on an amino acid multiple sequence alignment of the splicing blocks (A, B, F, G) using the ML method and evaluated with SH-aLRT. The substitution model, LG+G, was selected using ProtTest 3 (https://github.com/ddarriba/prottest3). Tree follows the same formatting as in panel A. ML, maximum likelihood; NJ, neighbor-joining; Prp8, pre-mRNA processing factor 8; SH-aLRT, Shimodaira–Hasegawa nonparametric approximate likelihood-ratio test. (TIF) [file pbio.3000104.s004.tif]

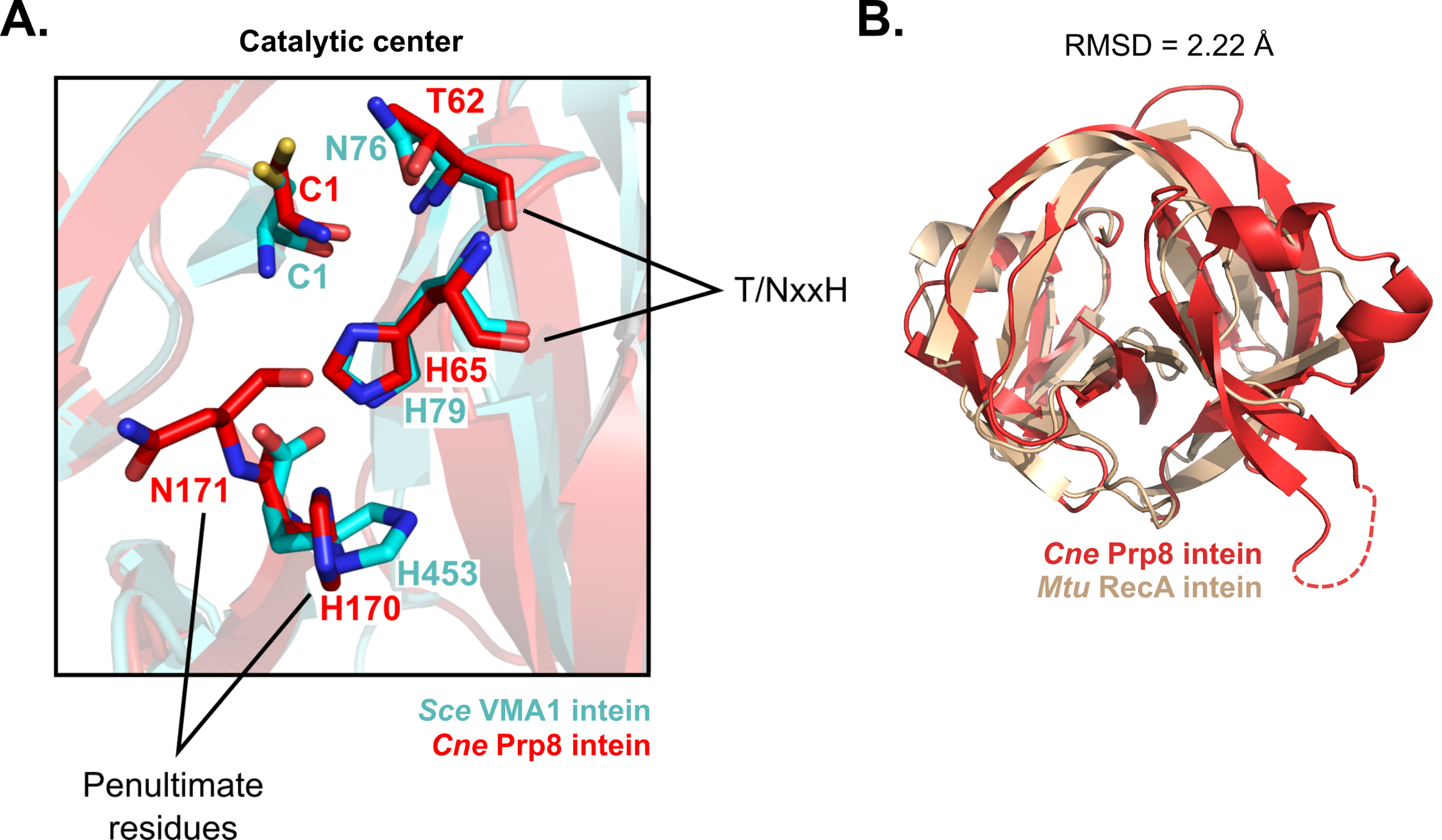

Supplement: S5 Fig — (A) Overlay of the Sce VMA1 intein and Cne Prp8 intein active sites. The Sce VMA1 intein (cyan, PDB 1GPP) was overlaid with the Cne Prp8 intein (red). The active site residues, crucial to protein splicing, are shown as sticks and labeled. A majority of these conserved residues overlap exactly, such as the catalytic C1, and the Block B TxxH motif. The Sce VMA1 intein uses an asparagine (N76) rather than threonine in the TxxH motif, but the positioning is similar to the threonine (T62) of the Cne Prp8 intein. The penultimate histidines (H170 and H453) are in comparable positions except for the side chains, whose chi angles are different by 45°. The Sce VMA1 intein was not solved with the terminal asparagine. (B) Structural comparison of bacterial Mtu RecA intein and fungal Cne Prp8 intein. Overlay of the Mtu RecA intein (brown, PDB 2IMZ), and the Cne Prp8 intein (red) reveals structural similarities in major intein features, such as the anti-parallel β-sheet folding, that contribute to the horseshoe shape. The Hint domain, comprised of splicing Blocks A, B, F, and G, are generally aligned between the 2 inteins. The structures deviate at sequences between Blocks B and F, where the Cne Prp8 intein encoded a linker or endonuclease domain. The 2 structures have an RMSD value of 2.22 Å. Cne, C. neoformans; Mtu, Mycobacterium tuberculosis; PDB, Protein Data Bank; Prp8, pre-mRNA processing factor 8; RMSD, Root-mean-square deviation; Sce, Saccharomyces cerevisiae (TIF) [file pbio.3000104.s005.tif]

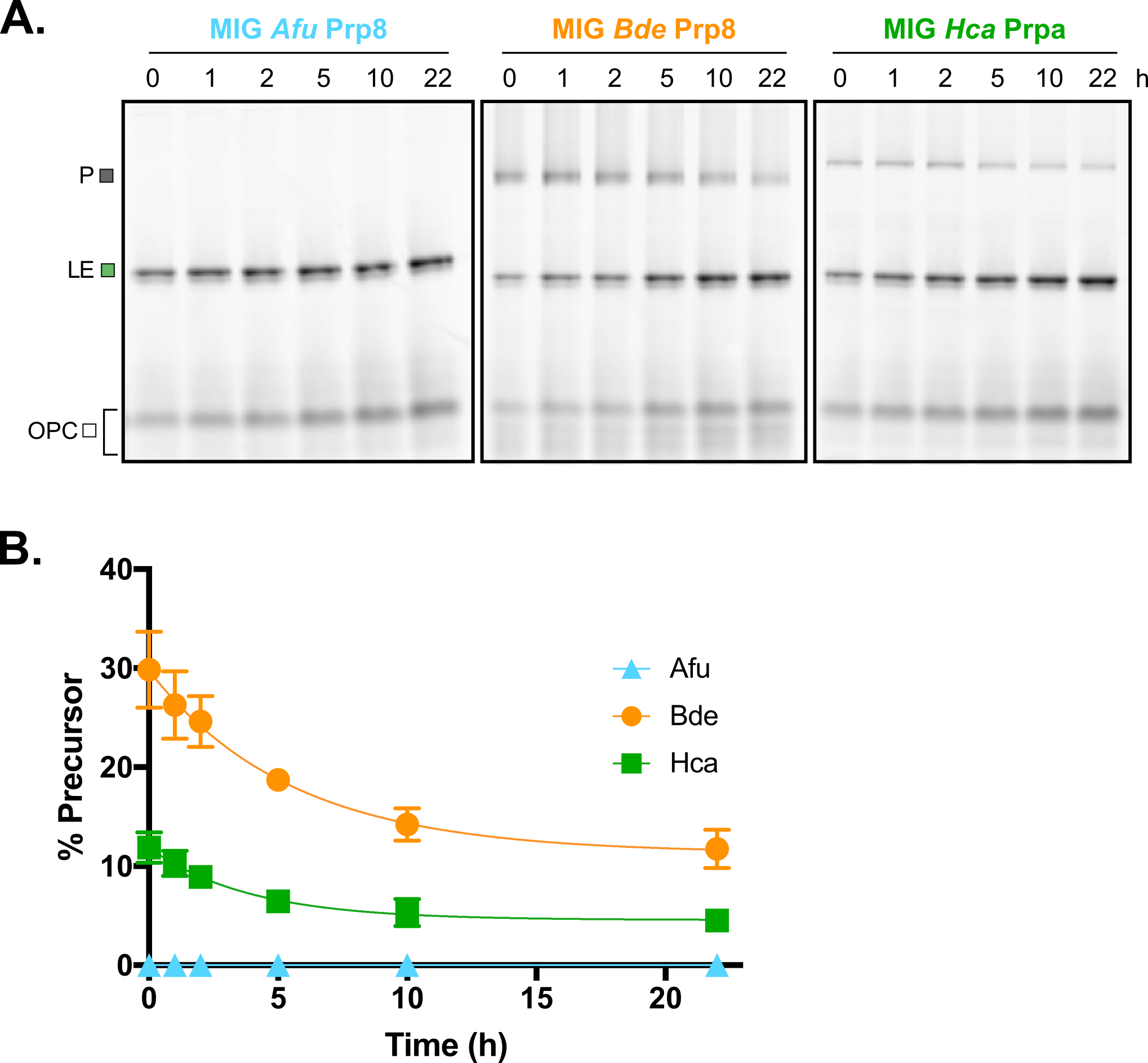

Supplement: S6 Fig — (A) Diverse Prp8 intein splicing patterns. Several Prp8 inteins from other fungal pathogens Afu, Bde, and Hca were cloned into MIG. Splicing was observed over time by the loss of precursor (P) and increase in LE, or simply by the presence of ligated exteins (for Afu). The gel shows that not all Prp8 inteins splice similarly, despite being placed in an identical extein context. (B) Precursor amounts vary greatly. A quantitation of precursor (P) at each time point shows that these Prp8 inteins are active but splice at variable rates. The Afu Prp8 intein is almost entirely spliced at the start of the assay (0 h), whereas Bde has 31% precursor at 0 h and Hca has 14% precursor at 0 h. Initial splicing rates were determined by calculating the loss of precursor over time (Pt0−Pt1/60 min) with standard error for MIG Bde Prp8 and MIG Hca Prp8, and are (5.9 ± 0.4) × 10−2% per min and (2.7 ± 0.9) × 10−2% per min, respectively. This suggests intein-mediated control of protein splicing. Data are representative of 3 biological replicates and mean standard deviations are shown. Trend lines are fit to show the decay curve. Data available in S1 Data. Afu, Aspergillus fumigatus; Bde, Batrachochytrium dendrobatidis; Hca, Histoplasma capsulatum; LE, ligated exteins; MIG, MBP-Intein-GFP; Prp8, pre-mRNA processing factor 8 (TIF) [file pbio.3000104.s006.tif]

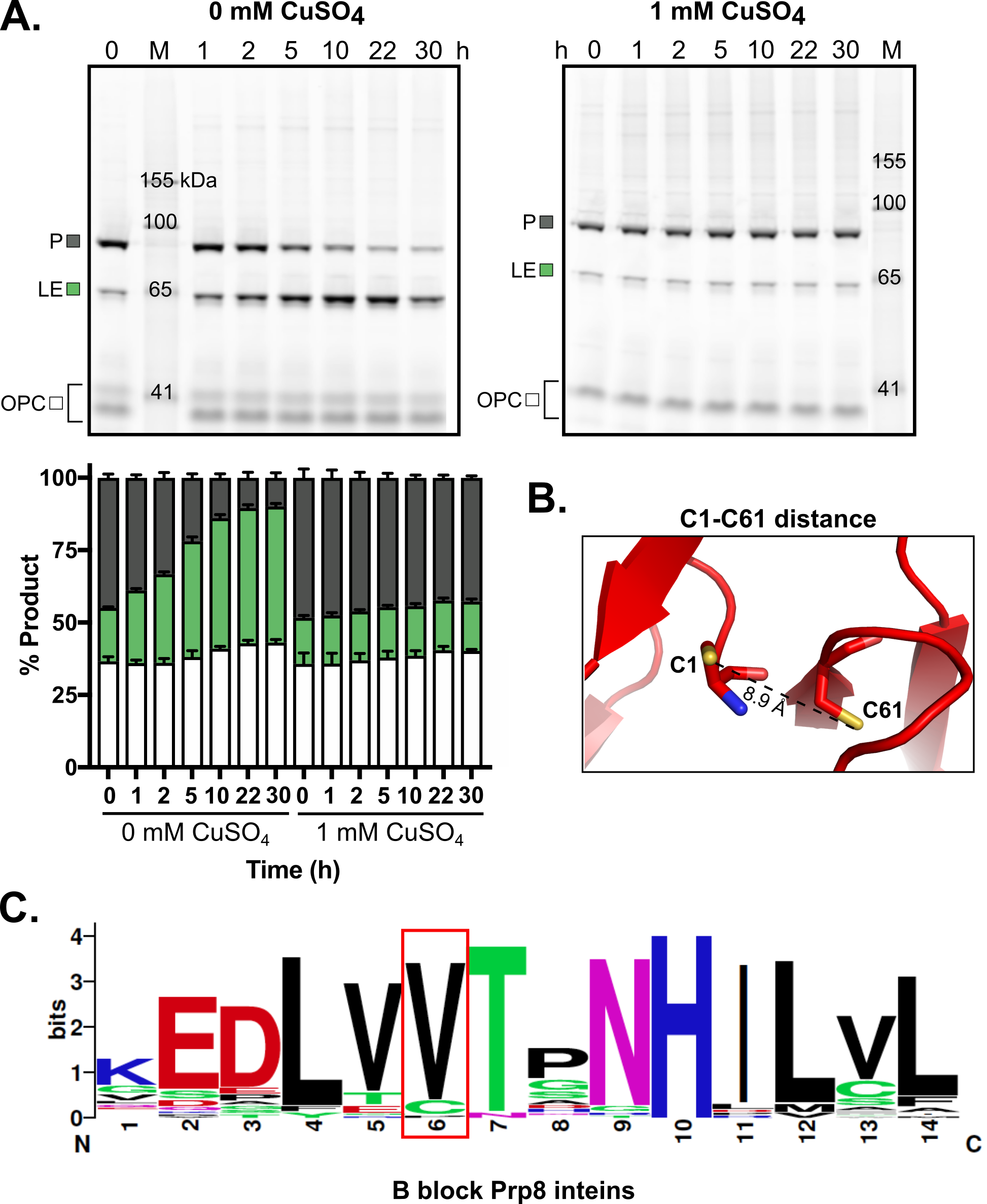

Supplement: S7 Fig — (A) Copper treatment causes inhibition. Induced MIG Prp8 A-1V cells were lysed and treated with 0 or 1 mM CuSO4. The lysates were incubated for the indicated time at 30°C and then frozen. Samples were separated on SDS-PAGE and scanned for GFP fluorescence. In the absence of copper, MIG Prp8 A-1V spliced well over 30 h, converting P into LE. There was little to no conversion of P to LE over time with copper addition. Quantitation is shown below in a stacked plot. Data are representative of 3 biological replicates and mean standard deviations are shown. Data available in S1 Data. (B) Relative position of 2 cysteines. There are only 2 cysteines present in the Cne Prp8 intein. Using the solved structure, a measurement of the distance between C1 and C61 (shown as sticks) was calculated to be 8.9 Å. (C) Valine is the preferred residue at position 61. A sequence logo was constructed of Block B from the 50 representative Prp8 inteins (S1 Fig). This shows absolute conservation of the histidine (position 10) and a strong preference for threonine (position 7) in the TxxH motif. However, the Block B cysteine (position 6, red box) is not highly conserved across Prp8 inteins, and most encode valine at this site. Cne, C. neoformans; GFP, green fluorescent protein; LE, ligated exteins; MIG, MBP-Intein-GFP; P, precursor; Prp8, pre-mRNA processing factor 8 (TIF) [file pbio.3000104.s007.tif]

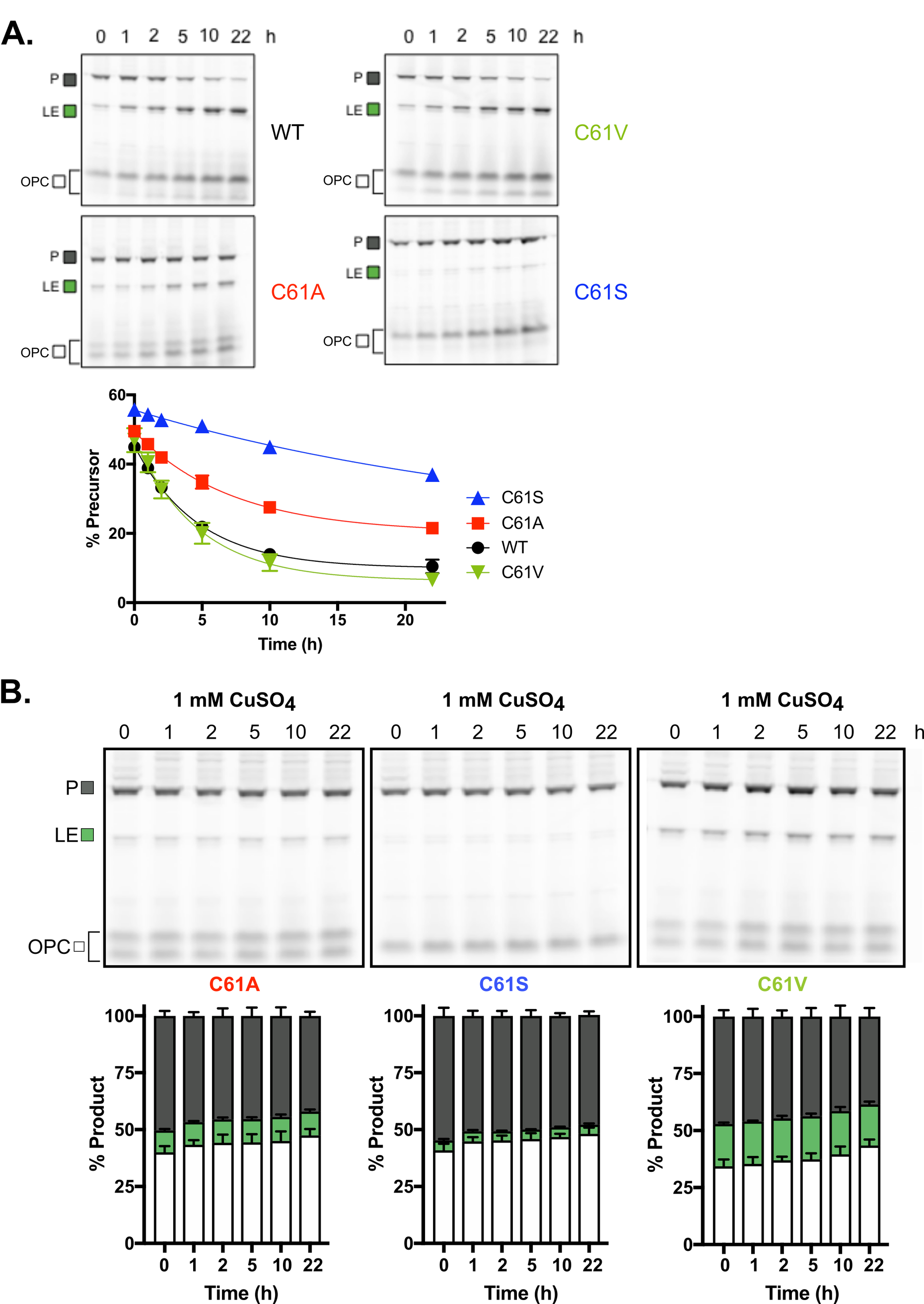

Supplement: S8 Fig — (A) Mutations to C61 in MIG Prp8 A-1V slow down splicing. The B block C61 was mutated to valine (C61V), alanine (C61A), and serine (C61S), and splicing was observed over time in MIG. Initial splicing rates were determined by calculating the loss of precursor over time (Pt0−Pt1/60 min) with standard error and are as follows: WT, (1.01 ± 0.07) × 10−1% per min; C61V, (1.07 ± 0.08) × 10−1% per min; C61A, (6.22 ± 0.50) × 10−2% per min, and C61S, (2.92 ± 1.04) × 10−2% per min. The C61V mutant splices similarly to WT, whereas C61A and C61S are slower. A quantitation is shown to the right with the amount of precursor (P) at each time point. Data are representative of 3 biological replicates and mean standard deviations are shown. Trend lines are fit to show the decay curve. Data available in S1 Data. (B) MIG Prp8 A-1V B block cysteine mutants are inhibited by copper. To test whether copper inhibition was caused by C1 oxidation, C61 mutants were treated with CuSO4. After induction of MIG, the cells were lysed, and 1 mM CuSO4 was added. The lysates were incubated at 30°C, and aliquots were collected at the indicated time. Samples were run on SDS-PAGE and scanned for GFP fluorescence. None of the C61 mutants show an increase in LE over time, with little loss of precursor (P). This indicates that at least C1 oxidation by copper is sufficient to cause the observed splicing inhibition and that disulfide bonds are not involved. Quantitation is shown below in a stacked plot. Data are representative of 3 biological replicates, and mean standard deviations are shown. Data available in S1 Data. GFP, green fluorescent protein; LE, ligated exteins; MIG, MBP-Intein-GFP; Prp8, pre-mRNA processing factor 8; WT, wild type. (TIF) [file pbio.3000104.s008.tif]

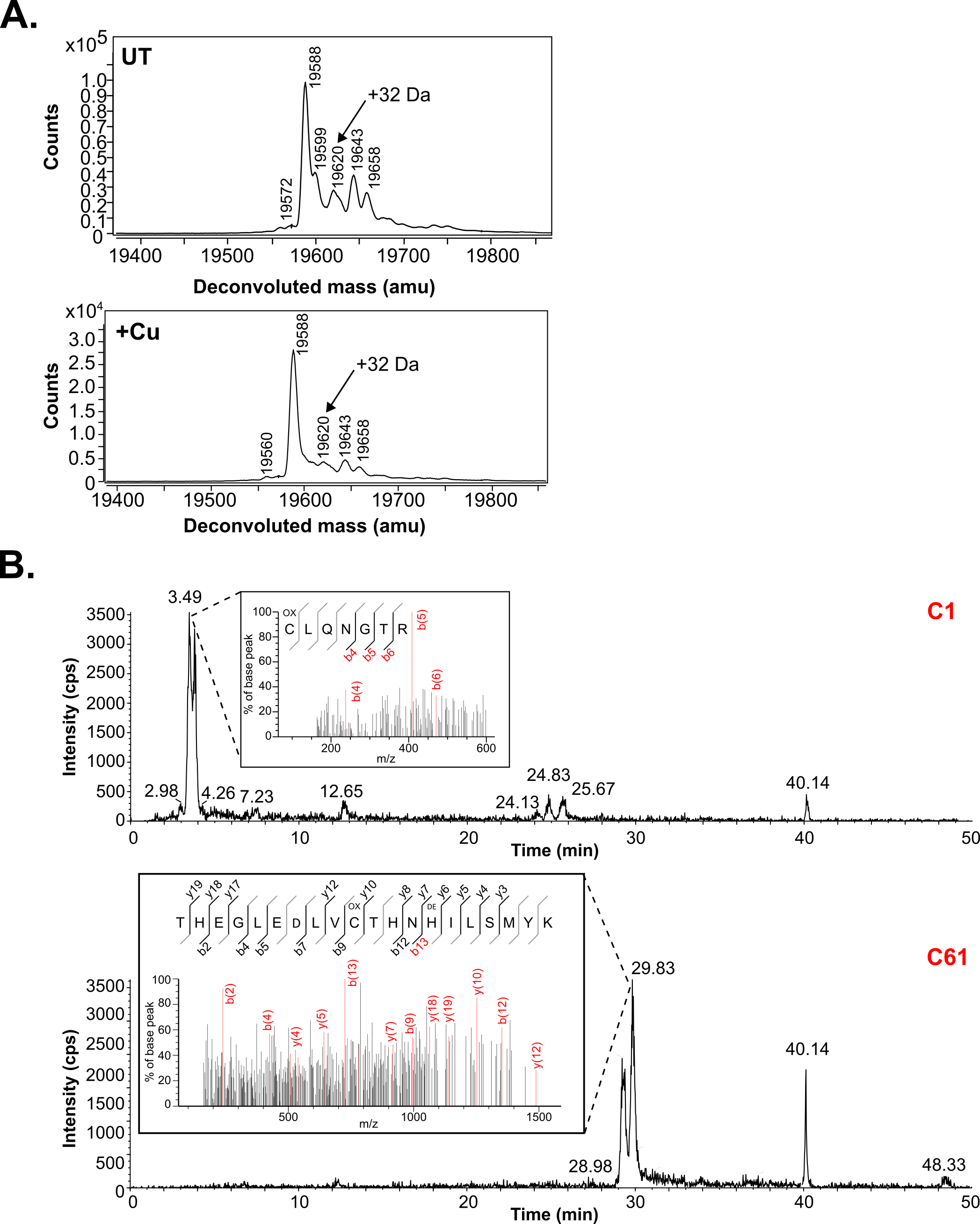

Supplement: S9 Fig — (A) Intact Cne Prp8 intein shows small mass shift. Purified Cne Prp8 intein was untreated or treated with 10× excess copper and separated and analyzed using LC-MS. The peaks were deconvoluted, and the expected mass of the Prp8 intein, 19,588 Da, is seen as the largest peak. A small, 32 Da shift (19,620 Da) was visible with both no treatment and copper treatment only (arrow). This suggests that highly reactive cysteines are modified by atmospheric oxygen alone. (B) C1 and C61 are oxidized with copper treatment. Trypsin-digested fragments of copper-treated Cne Prp8 intein were separated and sprayed using LC-MS/MS (insets). Peptides (red peaks) containing C1 or C61 were detected and further analyzed using multiple reaction MIDAS to confirm the identity and location of oxidation. The chromatogram shows elution time for both cysteines consistent with a single additional oxygen or a sulfenic acid modification. Cne, C. neoformans; LC-MS, liquid chromatography-mass spectrometry; LC-MS/MS, liquid chromatography-mass spectrometry/mass spectrometry; MIDAS, monitoring-initiated detection and sequencing; Prp8, pre-mRNA processing factor 8 (TIF) [file pbio.3000104.s009.tif]

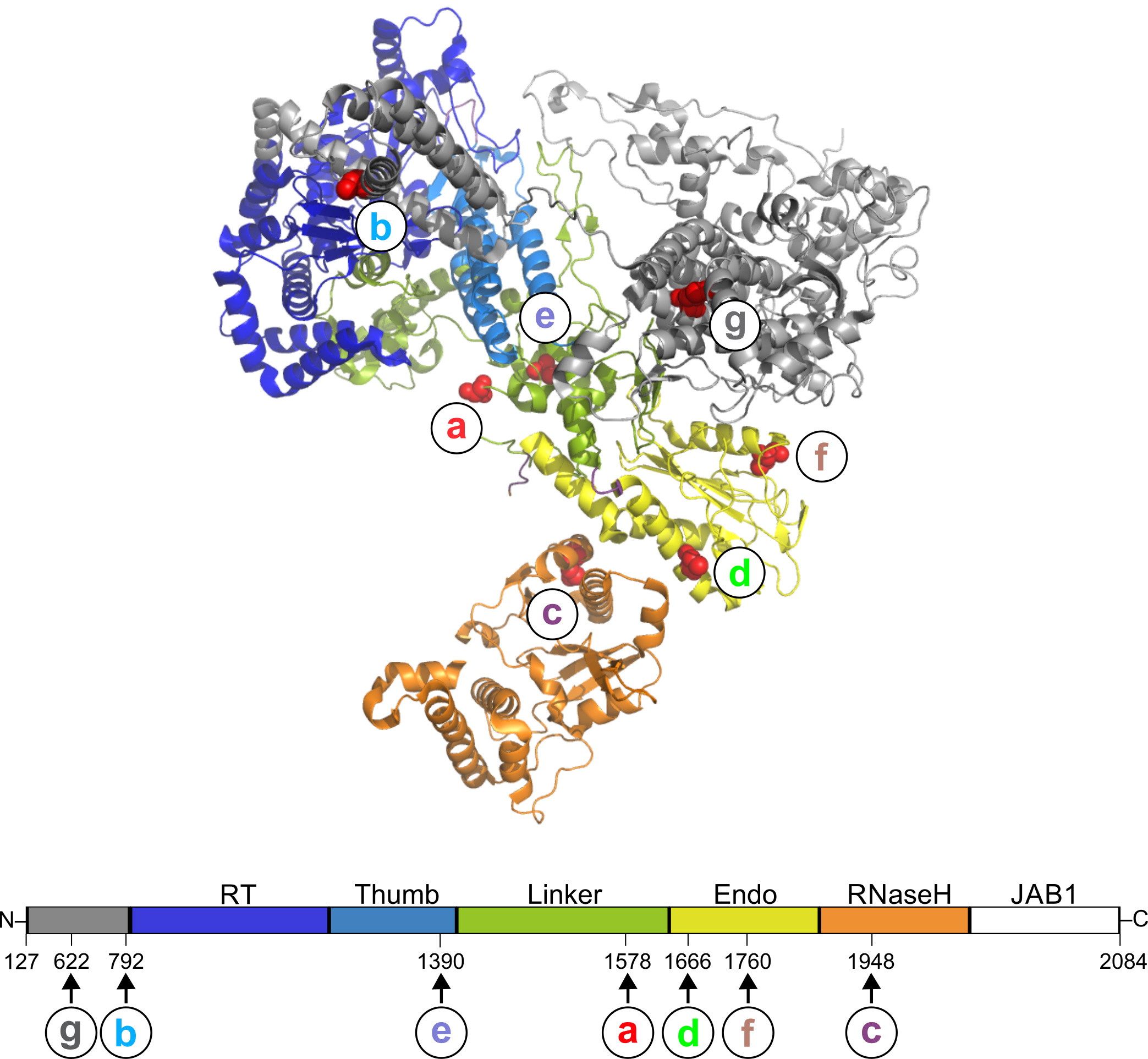

Supplement: S10 Fig — The 7 unique insertion sites (a–g) were mapped to a solved structure of Prp8 from a S. cerevisiae C complex spliceosome (PDB 5GMK, chain A from Wan and colleagues, 2016) by locating the +1 residue. This Prp8 structure was used because the insertion sites are all resolved. The +1 residues are shown as red spheres and labeled a through g. Most Prp8 inteins localize close to the active center of Prp8. Some insertions are in the N-terminal domain, which provides structural integrity to the spliceosome. A corresponding line diagram of Prp8 exteins shows the domains of the host protein from amino acid residues 127 to 2084 with arrows indicating the site of intein insertion with the residue number and insertion site letter. The domains are as follows: N-terminal domain, gray; RT Palm/Finger, dark blue; Thumb/X, light blue; linker, green; endonuclease, yellow; and RNase H-like, orange. PDB, Protein Data Bank; Prp8, pre-mRNA processing factor 8 (TIF) [file pbio.3000104.s010.tif]

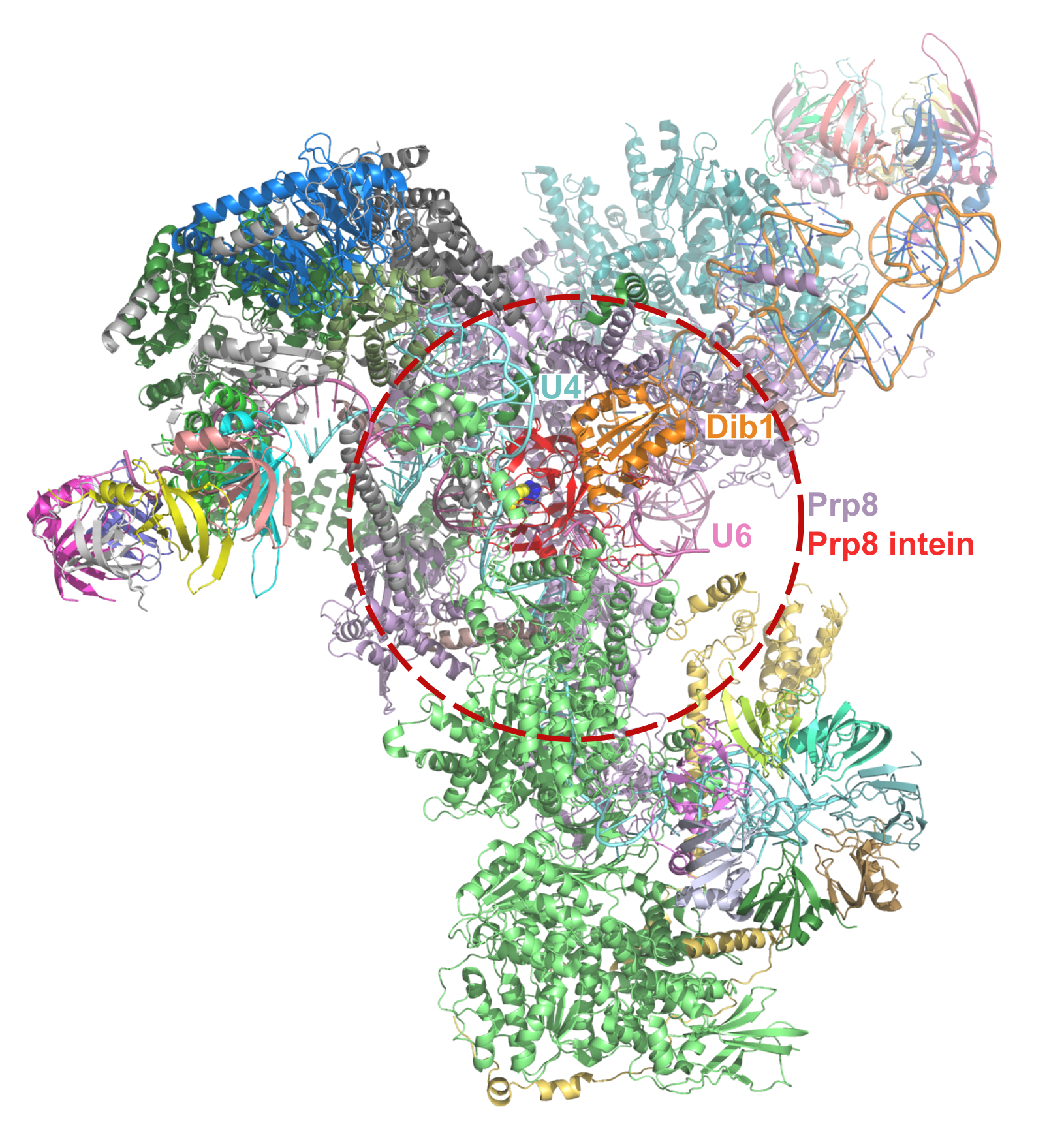

Supplement: S11 Fig — The Prp8 intein-containing Prp8 precursor model was docked into a cryo-EM tri-snRNP structure from Sce (PDB 5GAN) to look for intein-spliceosome disruptions. Prp8 is shown as lavender, and the Prp8 intein is shown as red, and the rest of the tri-snRP components are colored by chain. This reveals that the Prp8 intein would occupy a relatively crowded, centralized location of the tri-snRNP (circled). The intein clashes are shown here (with labels) and noted in Fig 7B. Cne, C. neoformans; cryo-EM, cryogenic electron microscopy; PDB, Protein Data Bank; Prp8, pre-mRNA processing factor 8; Sce, S. cerevisiae; tri-snRNP, triple small nuclear ribonucleoprotein. (TIF) [file pbio.3000104.s011.tif]
