# Supplementary material for: Spliceosomal Prp8 intein at the crossroads of protein and RNA splicing
Source: PLoS Biol. 2019 Oct 10;17(10):e3000104. doi: 10.1371/journal.pbio.3000104 (PMC6805012; doi:10.1371/journal.pbio.3000104)
Supplement: S1 Table — A list of bacterial strains used for various cloning, overexpression, and purification studies is provided. Strains of fungi and yeast used for in vivo studies are also listed. (DOCX) [file pbio.3000104.s012.docx]

**S1 Table** Bacterial and fungal strains

| **Strain** | **Features and comments** | **Source** |
| --- | --- | --- |
| DH5α | *E. coli* F^–^ *endAI recAl hsdRl7* (rK^-^ mK^-^) *deoR supE44 thi-J gyrA96 relA* | Gibco-BRL |
| MG1655(DE3) | *E. coli* F^–^ (λDE3) *ilvG rfb50 rph*1 | James Imlay |
| BL21(DE3) | *E. coli* F^–^ *ompT* *hsd*S_B_(r_B_^–^ m_B_^–^) *gal dcm* (λDE3) | Novagen |
| H99 | Wild type strain of *C. neoformans* H99 used to construct Prp8ΔIn | Joseph Heitman |
| Prp8ΔIn | Inteinless strain of *C. neoformans* H99, whereby *PRP8* lacking the intein is expressed from the Safe Haven site and the native *PRP8* locus is replaced by a *HYG* cassette. | Present study |
| S288C | Wild type strain of *S. cerevisiae* used for Western blotting as a strain lacking the Prp8 intein | Robert K. Mortimer |
