# Supplementary material for: Spliceosomal Prp8 intein at the crossroads of protein and RNA splicing
Source: PLoS Biol. 2019 Oct 10;17(10):e3000104. doi: 10.1371/journal.pbio.3000104 (PMC6805012; doi:10.1371/journal.pbio.3000104)
Supplement: S2 Table — A list of MIG constructs and purification vectors with corresponding backbones are provided. MIG, MBP-Intein-GFP. (DOCX) [file pbio.3000104.s013.docx]

**S2 Table** Plasmids and constructs

| **Plasmid/construct** | **Features and comments** | **Source** |
| --- | --- | --- |
| pACYCDuet-1 | Expression vector, T7 promoter, Cam^R^. | Novagen |
| pACYC MIG SufB | Used as cloning backbone by removing *M. tuberculosis* SufB intein insert using ClaI/SphI. | Topilina et al. 2015 |
| pACYC MIG Prp8 | *C. neoformans* var. *grubii* H99 Prp8 intein flanked by short native exteins (N-extein: FWEKA; C-extein: SGFEE) cloned into ClaI/SphI sites between MBP and GFP coding sequences in pACYCDuet-1 backbone. | Present study |
| pACYC MIG Prp8 A-1V | Same as pACYC MIG Prp8 but with the indicated amino acid mutation at the last amino acid of the N-extein (position -1). | Present study |
| pACYC MIG Prp8 A-1V C61A/S/V | Same as pACYC MIG Prp8 A-1V but with the indicated amino acid mutation at the B block C61. | Present study |
| pACYC MIG *Afu* Prp8 | *Aspergillus fumigatus* 293 Prp8 intein flanked by short native exteins (N-extein: FWERA; C-extein: SGFEE) cloned into ClaI/SphI sites between MBP and GFP coding sequences in pACYCDuet-1 backbone. | Present study |
| pACYC MIG *Bde* Prp8 | *Batrachochytrium dendrobatidis* JEL423 Prp8 intein flanked by short native exteins (N-extein: FWEKA; C-extein: SGFEE) cloned into ClaI/SphI sites between MBP and GFP coding sequences in pACYCDuet-1 backbone. | Present study |
| pACYC MIG *Hca* Prp8 | *Histoplasma capsulatum* G186A Prp8 intein flanked by short native exteins (N-extein: FWERA; C-extein: SGFEE) cloned into ClaI/SphI sites between MBP and GFP coding sequences in pACYCDuet-1 backbone. | Present study |
| pET28a | Expression vector, T7 promoter, C-terminal His_6_ tag, thrombin cleavage, Kan^R^. | Novagen |
| pET28a *Cne* Prp8 intein | *C. neoformans* var. *grubii* H99 Prp8 intein with 2 native N-extein residues (KA) in pET28a backbone at NcoI/XhoI sites. | Present study |
| pET47b | Expression vector, T7 promoter, N-terminal His_6_ tag, PreScission cleavage, Kan^R^. | Novagen |
| pET47b *Cne* Prp8 intein 3 N-exteins | *C. neoformans* var. *grubii* H99 Prp8 intein with 3 native N-extein residues (EKA) in pET47b backbone at BamHI/NotI sites. | Present study |
