# Supplementary material for: Spliceosomal Prp8 intein at the crossroads of protein and RNA splicing
Source: PLoS Biol. 2019 Oct 10;17(10):e3000104. doi: 10.1371/journal.pbio.3000104 (PMC6805012; doi:10.1371/journal.pbio.3000104)
Supplement: S3 Table — Primers used for the construction of various vectors or for the mutation of plasmids are provided. (DOCX) [file pbio.3000104.s014.docx]

**S3 Table** Oligonucleotide primers

| **Oligo ID** | **Sequence (5′ to 3′)** | **Application** |
| --- | --- | --- |
| IDT3967 | gggGCATGCaTTCTGGGAGAAAGCCTGTCTGCAG | *Cne* Prp8 intein forward primer for MIG with SphI site. |
| IDT3968 | gggATCGATTTCCTCAAATCCTGAGTTGTGCAGTACC | *Cne* Prp8 intein reverse primer for MIG with ClaI site. |
| IDT4027 | accattctgcagacaNNStttctcccagaatgcatgcatgtaaggcc | MIG Prp8 N-1 random mutagenesis sense primer. N is any nucleotide, S is a G or C. |
| IDT4028 | ggccttacatgcatgcattctgggagaaaNNStgtctgcagaatggt | MIG Prp8 N-1 random mutagenesis antisense primer. N is any nucleotide, S is a G or C. |
| IDT5841 | GAGggactcgaagatctggtcgctacccataaccacatcctTTC | MIG Prp8 A-1V mutagenesis C61A sense primer. |
| IDT5840 | gaaaggatgtggttatgggtagcgaccagatcttcgagtccctc | MIG Prp8 A-1V mutagenesis C61A antisense primer. |
| IDT5987 | GAGGGACTCGAAGATCTGGTCAGTACCCATAACCACATCCTTTC | MIG Prp8 A-1V mutagenesis C61S sense primer. |
| IDT5986 | GAAAGGATGTGGTTATGGGTACTGACCAGATCTTCGAGTCCCTC | MIG Prp8 A-1V mutagenesis C61S antisense primer. |
| IDT6026 | TGAgggactcgaagatctggtcgttacccataaccacatccttTCT | MIG Prp8 A-1V mutagenesis C61V sense primer. |
| IDT6027 | AGAaaggatgtggttatgggtaacgaccagatcttcgagtcccTCA | MIG Prp8 A-1V mutagenesis C61V antisense primer. |
| IDT5893 | GAGGGAAGGCCTTACATGCATGCaTTTTGGGAAAGAGCATGCCTTG | *Afu* Prp8 intein forward primer for MIG with SphI site with overhang InFusion cloning. |
| IDT5894 | TCTCCTTTGCTCATATCGATTTCTTCAAATCCACTGTTATGCAAG | *Afu* Prp8 intein reverse primer for MIG with ClaI site with overhang for InFusion cloning. |
| IDT5891 | GAGGGAAGGCCTTACATGCATGCaTTTTGGGAAAAGGCATGTTTTGc | *Bde* Prp8 intein forward primer for MIG with SphI site with overhang InFusion cloning. |
| IDT5892 | TCTCCTTTGCTCATATCGATCTCCTCAAACCCAGAGTTATGC | *Bde* Prp8 intein reverse primer for MIG with ClaI site. |
| IDT5889 | GAGGGAAGGCCTTACATGCATGCaTTTTGGGAACGAGCCTGTCT | *Hca* Prp8 intein forward primer for MIG with SphI site with overhang InFusion cloning. |
| IDT5890 | TCTCCTTTGCTCATATCGATTTCTTCAAATCCGCTGTTATGT | *Hca* Prp8 intein reverse primer for MIG with ClaI site with overhang for InFusion cloning. |
| *Cne* Prp8 pET28a F | CTTTAAGAAGGAGATATACCATGGGCAAAGCCTGTCTGCAGAATGGTAC | *Cne* Prp8 intein with two native N-exteins (KA) forward primer for amplification and cloning into pET28a with NcoI end. |
| *Cne* Prp8 pET28a R | cagtggtggtggtggtggtgctcgagTGAGTTGTGCAGTACCAAATAGTC | *Cne* Prp8 intein with S+1 reverse primer for amplification and cloning into pET28a with XhoI end. |
| IDT6143 | gggGGATCCaGAGAAAGCCTGTCTGCAGAATG | *Cne* Prp8 intein with three native N-exteins (EKA) forward primer for amplification and cloning into pET47b with BamHI end. |
| IDT6142 | gggGCGGCCGCttaGTTGTGCAGTACCAAATAGTCATAAC | *Cne* Prp8 intein reverse primer for amplification and cloning into pET47b with NotI end. |
