# Supplementary material for: Spliceosomal Prp8 intein at the crossroads of protein and RNA splicing
Source: PLoS Biol. 2019 Oct 10;17(10):e3000104. doi: 10.1371/journal.pbio.3000104 (PMC6805012; doi:10.1371/journal.pbio.3000104)
Supplement: S4 Table — Data collection, refinement statistics, and model details for (A) the unbound and (B) the Zn2+-bound Cne Prp8 intein crystal structures. Cne, C. neoformans; Prp8, pre-mRNA processing factor 8 (DOCX) [file pbio.3000104.s015.docx]

**S4A Table** Data collection, refinement statistics, and model details for *Cne* Prp8 intein crystal structure

| ***Data Collection*** |  |
| --- | --- |
| Wavelength (Å) | 0.97946 |
| Resolution (Å) | 32.7-1.75 (1.81-1.75) |
| Space group | P 1 |
| Unit cell |  |
| a,b,c (Å) | 63.15 63.23 80.20 |
| α,β, (°) | 103.32 95.53 117.34 |
| Total reflections | 211,948 (19,137) |
| Unique reflections | 90,641 (8,259) |
| Multiplicity | 2.3 (2.3) |
| Completeness (%) | 85.91 (77.71) |
| Mean *I/σ(I)* | 12.48 (1.55) |
| *R_merge_ (%)* | 0.069 (0.91) |
|  |  |
| ***Refinement*** |  |
| *R_work_* | 0.197 (0.293) |
| *R_free_* | 0.235 (0.310) |
| No. of non-H atoms | 7576 |
| macromolecules | 7026 |
| solvent | 550 |
| RMS (bonds) (Å) | 0.005 |
| RMS (angles) (°) | 1.02 |
| Ramachandran |  |
| favored (%) | 98.42 |
| allowed (%) | 1.58 |
| outliers (%) | 0.00 |
| Rotamer outliers (%) | 0.00 |
| Clashscore | 3.45 |
| Average B-factor (Å^2^) | 30.39 |
| macromolecules | 29.81 |
| solvent | 37.80 |

Statistics for the highest-resolution shell are shown in parentheses.

**S4B Table** Data collection, refinement statistics, and model details for *Cne* Prp8 intein crystal structure with Zn^2+^.

| ***Data Collection*** |  |
| --- | --- |
| Wavelength (Å) | 0.97950 |
| Resolution (Å) | 40.09-1.84 (1.91-1.84) |
| Space group | P 1 |
| Unit cell |  |
| a,b,c (Å) | 63.09 63.04 78.64 |
| α,β, (°) | 94.86 103.13 117.44 |
| Total reflections | 168,564 (12,998) |
| Unique reflections | 71,327 (6,013) |
| Multiplicity | 2.4 (2.2) |
| Completeness (%) | 80.43 (67.61) |
| Mean *I/σ(I)* | 9.38 (1.59) |
| *R_merge_ (%)* | 0.084 (0.47) |
|  |  |
| ***Refinement*** |  |
| *R_work_* | 0.199 (0.273) |
| *R_free_* | 0.243 (0.319) |
| No. of non-H atoms | 7,645 |
| macromolecules | 7,074 |
| Zn | 9 |
| solvent | 562 |
| RMS (bonds) (Å) | 0.006 |
| RMS (angles) (°) | 0.89 |
| Ramachandran |  |
| favored (%) | 97.69 |
| allowed (%) | 2.19 |
| outliers (%) | 0.12 |
| Rotamer outliers (%) | 0.13 |
| Clashscore | 5.15 |
| Average B-factor (Å^2^) | 25.07 |
| macromolecules | 24.47 |
| Zn | 75.71 |
| solvent | 31.84 |

Statistics for the highest-resolution shell are shown in parentheses.
